# Natural Products as Chemopreventive Agents by Potential Inhibition of the Kinase Domain in ErbB Receptors

**DOI:** 10.3390/molecules22020308

**Published:** 2017-02-17

**Authors:** Maria Olivero-Acosta, Wilson Maldonado-Rojas, Jesus Olivero-Verbel

**Affiliations:** Environmental and Computational Chemistry Group, School of Pharmaceutical Sciences, Zaragocilla Campus, University of Cartagena, Cartagena 130015, Colombia; moliveroa@unicartagena.edu.co (M.O.-A.); wmaldonador@unicartagena.edu.co (W.M.-R.)

**Keywords:** natural compounds, molecular docking, HER receptors, AutoDock Vina

## Abstract

Small molecules found in natural products provide therapeutic benefits due to their pharmacological or biological activity, which may increase or decrease the expression of human epidermal growth factor receptor (HER), a promising target in the modification of signaling cascades involved in excessive cellular growth. In this study, in silico molecular protein-ligand docking protocols were performed with AutoDock Vina in order to evaluate the interaction of 800 natural compounds (NPs) from the NatProd Collection (http://www.msdiscovery.com/natprod.html), with four human HER family members: HER1 (PDB: 2ITW), HER2 (PDB: 3PP0), HER3 (PDB: 3LMG) and HER4 (PDB: 2R4B). The best binding affinity values (kcal/mol) for docking pairs were obtained for HER1-podototarin (−10.7), HER2-hecogenin acetate (−11.2), HER3-hesperidin (−11.5) and HER4-theaflavin (−10.7). The reliability of the theoretical calculations was evaluated employing published data on HER inhibition correlated with in silico binding calculations. IC_50_ values followed a significant linear relationship with the theoretical binding Affinity data for HER1 (*R* = 0.656, *p* < 0.0001) and HER2 (*R* = 0.543, *p* < 0.0001), but not for HER4 (*R* = 0.364, *p* > 0.05). In short, this methodology allowed the identification of several NPs as HER inhibitors, being useful in the discovery and design of more potent and selective anticancer drugs.

## 1. Introduction

The epidermal growth factor receptor, EGFR, is a transmembrane receptor tyrosine kinase (RTK), known to be overexpressed in approximately 90% of tumors [1] that strongly affects the quality of life and outcomes of cancer patients all over the world [2]. It is currently affiliated to the ErbB/HER family of receptors which comprises of four structurally related signaling oncoproteins HER1 (EGFR/ErbB1), HER2 (ErbB2/Neu), HER3 (ErbB3), and HER4 (ErbB4) [3]. The receptor is composed of different elements, such as a glycosylated extracellular domain, a single hydrophobic transmembrane segment, an intracellular portion with a regulatory juxtamembrane segment, a carboxy terminal tail composed of specific tyrosine-containing sequences and a key protein kinase domain [4,5].

These receptors play a major role in the modulation of diverse cellular functions, such as cell proliferation, differentiation, adhesion, survival, motility [3] and programmed cell death [6] that are often dysregulated, generating a cascade of responses able to promote the formation and progression of various malignancies such as: glioblastoma [7], muscle invasive bladder cancer [8], non-small-cell lung cancer [9], squamous cell carcinoma of the skin [10], gastric cancer [11] and breast cancer [12], via aberrant overexpression, kinase activation and mutation. Studies have demonstrated that some of these receptors have special characteristics, such as HER2 and HER3; HER2 possesses no known natural ligands [13,14] and HER3, the only member of the HER family lacking intrinsic tyrosine kinase activity [15].

The activation of HER receptors is the result of their ability to bind effectively to different ligands, like epidermal growth factor (EGF), transforming growth factor-α (TGFA), heparin-binding EGF-like growth factor, amphiregulin, betacellulin, epiregulin and the latest addition to the mammalian family of EGFR, epigen [16], either specifically or to one or more receptors. After ligand binding to the extracellular domain of the receptor, a conformational change takes place in form of functionally active dimers (EGFR-EGFR (homodimer) or EGFR-HER2, EGFR-HER3, EGFR-HER4 (heterodimer)). This allows the transphosphorylation on tyrosine residue to induce and enhance the activation of the intrinsic tyrosine kinase domain [3], process that ultimately modifies the behavior of a normal cell through the output of signals for cellular proliferation, anti-apoptosis, angiogenesis and metastasis [17].

Given the prominent importance of EGFR signaling in cancer development, scientists have developed the following strategies: anti-EGFR monoclonal antibodies and small-molecule EGFR tyrosine kinase inhibitors (TKIs) [18]. Anti-EGFR antibodies, e.g., cetuximab and panitumumab, bind to the extracellular domain of EGFR monomer and compete against endogenous ligands by receptor binding, blocking ligand-induced receptor activation. Small molecule EGFR inhibitors, such as erlotinib, gefitinib and lapatinib, compete specifically with ATP for bind to the catalytic tyrosine kinase domain, inhibiting autophosphorylation processes and downstream signaling of cancer cells [18]. The inhibition of EGFR and its related proteins is considered as a standard approach in cancer investigations. Nevertheless, the existence of specific mutations in the receptor, restricts the efficacy of EGFR tyrosine kinase inhibitors (EGFR-TKIs). These, can make more effective treatments as seen in the example of the rare I744M exon 19 mutation of *EGFR*, which could predict dramatic responsiveness to TKI [19]; or the presence of the T790M gatekeeper mutation, located in the EGFR tyrosine kinase domain, which accounts for half of drugs resistance [20,21,22], posing a threat to the overall efficacy of this strategy.

In order to surpass and address the obstacles that can lead to the resistance mechanisms there is a growing need to find and develop new generations of EGFR-TKIs with broad kinase selectivity and desirable physicochemical properties that can in one way or another intervene in the blockage of EGFR mediated oncogenic function. A well-known approach is the use of natural products in response to the unmet medical needs described above. In this work, we suggest the application of computational tools, including molecular docking methods, pharmacophores, and web server ADME/Tox property calculator for the identification of lead compounds from small sized natural compounds (NatProd Collection), as potential chemopreventive agents that can be key to the discovery and development of new therapeutic drug candidates by modulation of the kinase domain of the ErbB (HER) receptors.

## 2. Results and Discussion

### 2.1. Structural Comparison of HER Receptors

**Figure 1:**
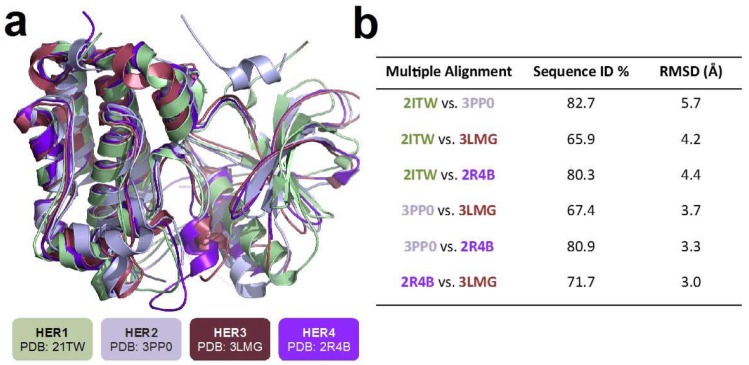
Superimposing of four human HER family structures (HER1-4) (**a**); Sequence identity and RMSD in angstrom (Å) for each HER Receptor (**b**).

The superposition of the 3D HER receptor structures (PDBs: 2ITW, 3PP0, 3LMG, 2R4B) as well as the RMSD values for each pair of them are presented in Figure 1. The three-dimensional structures of HER receptors have some differences. The comparative structural analysis considered in this work for the four human HER receptors (whole proteins), showed a sequence identity in a range between 65.9% and 82.47%, indicating structural differences, that were also observed for their 3D structures, with RMSD values ranging between 3.0 and 5.7 Å (Figure 1), being HER1 (2ITW) the one with the greatest spatial differences in terms of backbone alpha carbons (4.2–5.7 Å), compared with the other HER structures.

The multiple sequence alignment of the four HER receptor binding sites (41 amino acids), revealed major differences between evaluated protein structures. It showed percentage sequence identities between 57.6 % and 80.6 %, with RMSD values ranging from 1.81 to 4.41 Å. A lack of some important residues on the binding site, such as Asp for HER-3, can be observed in Figure S2b. This residue may influence the theoretical affinity and binding mode for evaluated natural compounds on HER structures.

### 2.2. Virtual Screening for Identification of Potential Inhibitors of HER Kinase Domain

Molecular docking validation performed by re-docking of co-crystallized ligand on the evaluated HER kinase domains (2ITW, 3PP0, 3LMG and 2R4B) revealed an optimal reproduction compared to experimental binding mode (co-crystallized), with satisfactory results, except for 2ITW, because the co-crystallized ligand undergoes fragmentation of the aromatic rings when it is docked on HER1 using AutoDock Vina; therefore, the results cannot be compared with the experimental pose. Moreover, RMSD cannot be calculated either (See Supplementary Materials Figure S3d). The best obtained RMSD values between predicted and experimental poses were 0.60 Å for HER2, 1.83 Å for HER3, and 0.95 Å for HER4 (See Supplementary Materials Figure S3a–c). Obtained results were in agreement with similar study conducted for this purpose [23].

**Figure 2:**
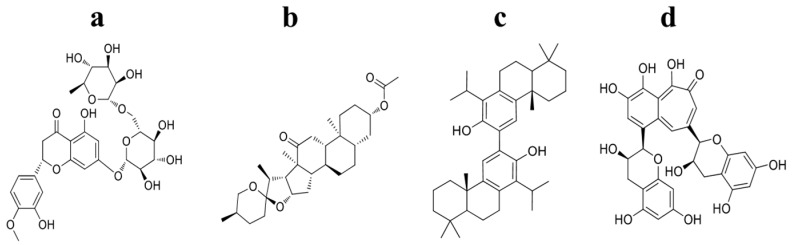
Four NPs selected as promissory HER Receptor inhibitors: (**a**) hesperidin; (**b**) hecogenin acetate; (**c**) podototarin; and (**d**) theaflavin.

A Virtual screening of the NPs found in the NatProd Collection with four kinase domains of HER receptors was performed by molecular docking with AutoDock Vina [24]. A thermodynamic potential, Gibbs free energy (kcal/mol), is considered in the protein-ligand binding process, suggesting an equilibrium state and complex stability when the value is more negative [25]. The results for the best ligands on each HER receptor are shown in Table 1. The NPs with the lowest binding affinities (kcal/mol) were selected for each evaluated proteins (Figure 2): HER1, podototarin (−10.7 kcal/mol); HER2, hecogenin acetate (−11.2 kcal/mol); HER3, hesperidin (−11.5 kcal/mol) and HER4, theaflavin (−10.7 kcal/mol). These NPs are considered potential HER inhibitors.

**Figure 3:**
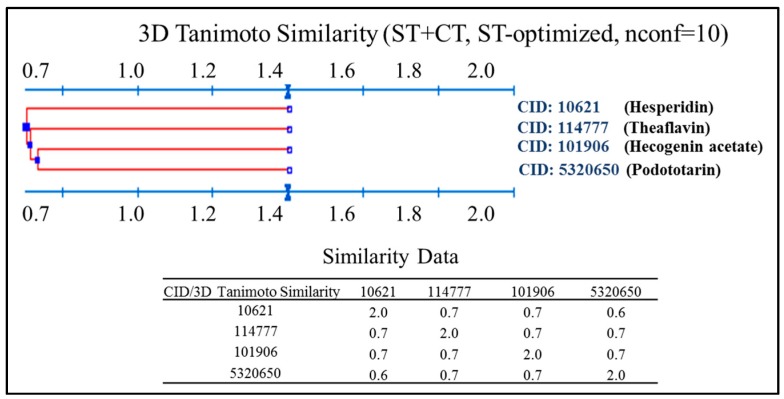
3D Clustering analysis for evaluated NPs.

A 3D Tanimoto similarity shape analysis was conducted for the virtual NP hits using the Structure Clustering tool in PubChem database (www.pubchem.ncbi.nlm.nih.gov). Compound identifiers (CID) were used as input for structure clustering tool. The dendogram shows that although these compounds have some degree of similarity (Figure 3), they are structurally different. These observations are somewhat predictable given to some critical differences on the binding site for each evaluated protein structure (PDBs: 2ITW, 3PP0, 3LMG, 2R4B).

### 2.3. Binding Mode Analysis and Interacting Residues for NPs on HER Kinase Domain Binding Site

**Figure 4:**
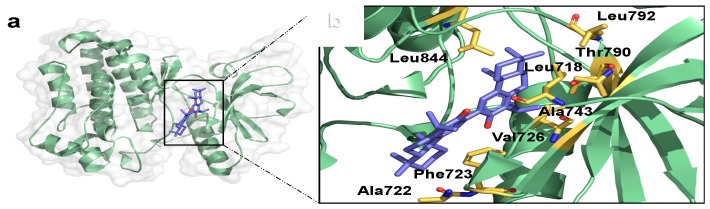

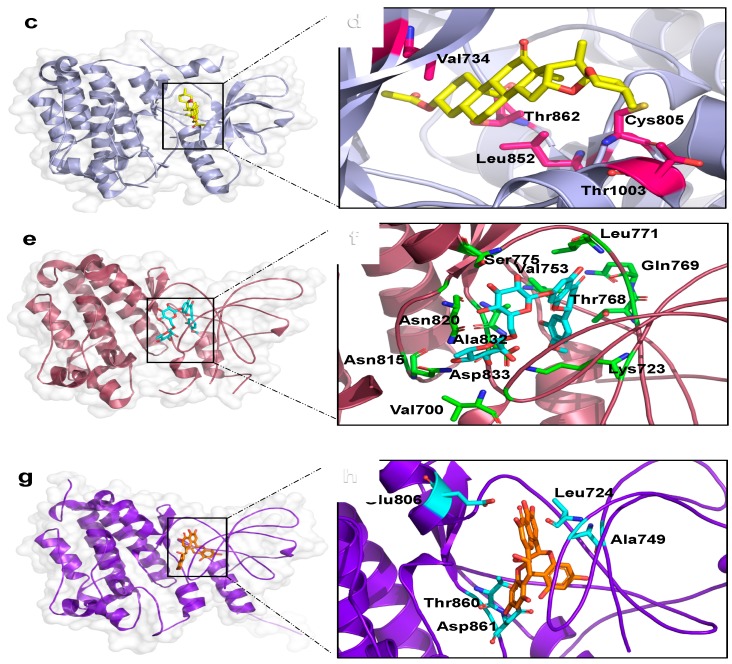
3D Structures for HER-NP complexes (left) and interacting residues on binding site (right). HER1 (2ITW)-podototarin (**a**,**b**); HER2 (3PP0)-hecogenin acetate (**c**,**d**); HER3 (3LMG)-hesperidin (**e**,**f**); HER4 (2R4B)-theaflavin (**g**,**h**).

**Figure 5:**
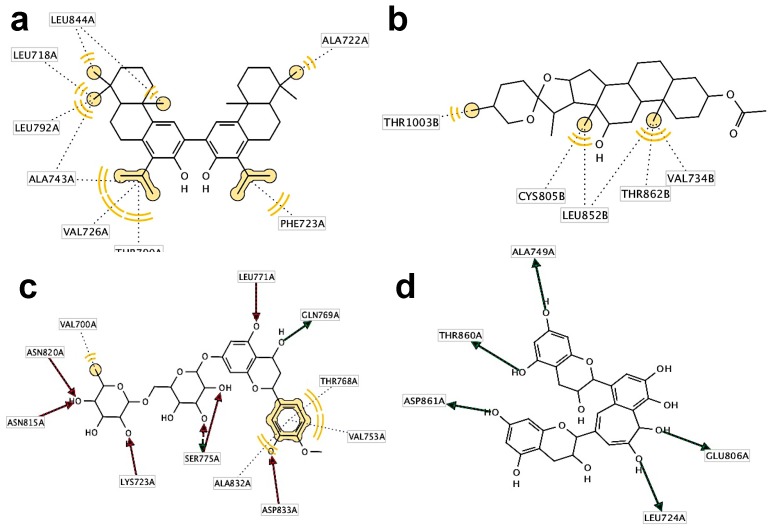
2D views of interacting residues predicted by LigandScout 3.1 for HER-NP complexes. HER1 (2ITW)-podototarin (**a**); HER2 (3PP0)-hecogenin acetate (**b**); HER3 (3LMG)-hesperidin (**c**); HER4 (2R4B)-theaflavin (**d**).

Best predicted protein-ligand complexes and interacting residues for the docking of the HER kinase domain with NPs are shown in Figure 4 and Figure 5, respectively. Podototarin in the HER1 binding site shows interactions with Val-726, Thr-790 and Ala-743 residues through hydrophobic interactions with isopropyl and methyl groups (Figure 4a,b). The interacting residues for the HER2—hecogenin acetate complex were Thr-1003, Cys-805, Leu-8528, Val-734 and Thr-862, presenting hydrophobic interactions with methyl groups (Figure 4c,d). In the case of hesperidin on HER3 binding site, hydrophobic interactions were predicted for amino acids Val-200 with a methyl group and Thr-768, Val-753 and Ala-832 with an aromatic ring; Lys-723, Asn-815, Asn-820, Ser-775, Leu-771 and Asp-833 residues form H-bonds through the hydroxyl groups (Figure 4e,f). Finally, predicted interacting residues with theaflavin on HER4 were Ala-749, Thr-860, Asp-861, Leu-724 and Glu-806, all of them through the hydrogen bond donor by their hydroxyl groups (Figure 4g,h).

### 2.4. Information Regarding the Selected NPs Is Described in the Following Section

Podototarin, also known as bitotarol, is a bisditerpenoid obtained from *Podocarpus totara*, a member of the Podocarpaceae family as well as from the heartwoods of *Podocarpus nivalis* and *P. Acutifolius* [26], distributed primarily in the southern hemisphere. It can be synthesized from (+)-totarol, a compound able to halt bacterial growth through by altering the cell division process [30]. To date there appears to be very little available information about its pharmacological effects.

Hecogenin acetate, a steroidal saponin found in plants of the genus Agave, possesses many particular qualities; for example, it exerts anti-cancer effects through modulation of the production of reactive species by inducing cell cycle arrest and senescence, and also by modulating ERK1/2 phosphorylation and MMP-2 production [31]. Furthermore, it has the capability to reduce inflammatory hyperalgesia, by mediation of opioid receptors and endogenous analgesic mechanisms in the descending pain-inhibitory branch in mice [27].

Hesperidin, is another a promising anti-cancer agent from Nature. There is evidence to support its capacity to induce cell death in various types of cancer such as: gastric, colon, breast, lung and liver, by various mechanisms [28]. It can alleviate cisplatin-induced hepatotoxicity in rats without affecting its antitumor activity [32], making it useful for the development of new concomitant therapies. It also possesses utility as an ulcer protective agent [33] and as an anti-depressant by mediation of Kappa opioid [34] and serotonergic 5HT1A receptors [35]. Recent studies show chemopreventive efficacy of hesperidin against chemically induced nephrotoxicity and renal carcinogenesis via amelioration of oxidative stress and modulation of multiple molecular pathways [36].

Theaflavins found in black teas are polyphenolic molecules known to have antioxidant effects. In recent studies, these have been shown to have in vitro anti-influenza and anti-inflammatory activity [37], protect nigral dopaminergic neurons [38], lower blood cholesterol [39] and induce apoptosis in human lung adenocarcinoma and esophageal carcinoma cells [40]. In addition, these compounds may be considered as potentially valuable supplementary therapeutic agents for prevention and treatment of *P. gingivalis*-associated periodontal diseases [29], showing also significant anti-herpes simplex virus type 1 (HSV-1) effect on A549 and Vero cell lines, by forbidding the entry of the virus to the host through blockage of adsorption and penetration processes [41].

### 2.5. Prediction of ADME-TOX Properties for Promisory Compounds Using FAF-Drugs3 Server

During the developmental stages of more effective medication candidates, it is likely to have various unsuccessful attempts at reaching a promissory compound for further study and clinical trials, on account of pharmacokinetic limitations, undesirable toxicities, and instabilities, properties that can be detected by using specialized programs, such as FAF-Drugs3 [42]. This server is able to predict ADME/Tox properties, such as absorption, distribution, metabolism, excretion and toxicity, as well as descriptors to ultimately reduce time, resources and costs on apparent promising candidates.

One of the filters applied ruled out the presence of Pan-assay Interfering compounds (PAINS) structures in the four NPs proposed as virtual hits, meaning that they do not appear as frequent hits (Table 2), which corroborates that these compounds are promising starting points for further exploration in cancer research

### 2.6. Molecular Docking Validation Using Reported Biological Data for Known HER Inhibitors

To determine if Affinity values calculated by AutoDock Vina could be utilized as indicators of the probability that a compound can behave as a HER receptor inhibitor, active compounds with confirmed inhibition activity, reported in PubChem BioAssay database, were docked to each receptor type (PDB: 2ITW, 3PP0, 3LMG, 2R4B). The PubChem chemical structure identifier (CID), biological activity (IC_50_), AutoDock Vina Affinity values, for these compounds, and the biological activity (Log IC_50_) are displayed in the Supplementary Material (Figures S5–S7).

**Figure 6:**
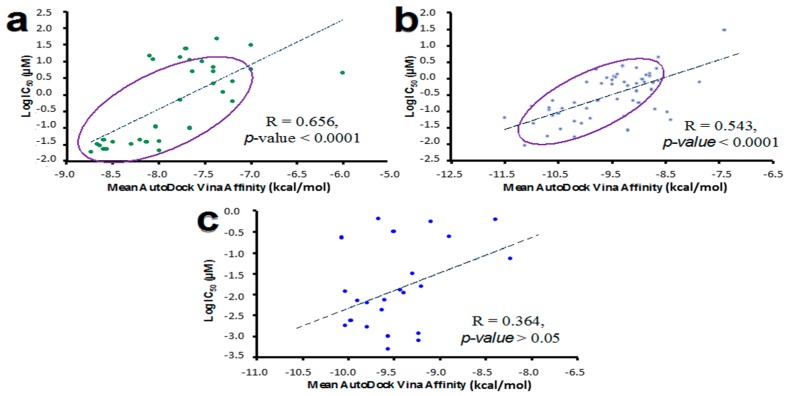
Correlation analysis between calculated Affinity values (kcal/mol) for known HER inhibitors and the logarithm of their half maximal inhibitory concentration (Log IC_50_). HER1, 2ITW (**a**); HER2, 3PP0 (**b**); and HER4, 2R4B (**c**). HER3 was not evaluated because no quantitative information was available for its inhibitors. It is known that HER3 lacks several residues that are critical for catalysis. However, it can serve as an activator of other EGFR family members [43].

The linear correlation analysis between binding and biological activity (IC_50_, µM, http://www.ncbi.nlm.nih.gov/pcassay). Affinity values calculated for known HER inhibitors are shown in Figure 6. IC_50_ values followed a linear relationship with the theoretical binding Affinity (kcal/mol) for HER1 (*R* = 0.656, *p* < 0.0001) and HER2 (*R* = 0.543, *p* < 0.0001), but not for HER4 (*R* = 0.364, *p* > 0.05). However, an inverse relationship between the Affinity absolute score (kcal/mol) versus the IC_50_ value is observed for the evaluated compound set (Figure 6c). This suggests that large Affinity absolute values for tested NPs will increase the probability of finding a compound to behave as HER inhibitor.

These results suggest that NPs displaying AutoDock Vina Affinity values lower than −10.7 kcal/mol can inhibit the kinase domain belonging to the HER receptors. These data support that NPs selected in this work (Figure 2) may act as chemopreventive agents in HER-mediated signaling.

## 3. Materials and Methods

### 3.1. Virtual Screening by Molecular Docking with AutoDock Vina

In order to identify natural compounds with the ability to interact with four HER receptors (HER1-4), a total of 800 optimized structures from NatProd Collection, MicroSource Discovery Systems (www.msdiscovery.com/natprod.html) were docked on four kinase domains belonging to each member of the HER receptor family. The Virtual Screening process was performed using AutoDock Vina program [24] from the Molecular Graphics Lab (La Jolla, CA, USA) at The Scripps Research Institute. The identification of natural compounds with promissory abilities to interact with the HER receptors was realized considering the Affinity absolute value (kcal/mol) as selection criteria. The binding modes for the best NPs docked on HER receptors were later analyzed employing LigandScout 3.1 program (Inte-Ligand, Vienna, Austria),by checking key features on the binding site, including the number, nature and type of existing interactions, such as hydrogen bonds, charge interactions and hydrophobic areas [44].

#### 3.1.1. Preparation and Selection of Crystallographic Structures of HER Kinase Domains for Molecular Docking with NPs

The four available HER kinase domain crystal structures (2ITW, 3PP0, 3LMG, 2R4B) were downloaded from RCSB Protein Data Bank database (www.rcsb.org/pdb/home/) in pdb formats file and were prepared with SYBYL-X 2.0 package for molecular docking evaluations. This process consisted of removing water molecules and co-crystalized ligands such as: 1,2,3,4-tetrahydrostaurosporine [45], 2-{2-[4-({5-chloro-6-[3-(trifluoromethyl)phenoxy]pyridin-3-yl}amino)-5*H*-pyrrolo[3,2-*d*]pyrimidin-5-yl]ethoxy}etanol [46], *N*-{3-chloro-4-[(3-fluorobenzyl)oxy]phenyl}-6-ethylthieno[3,2-*d*]pyrimidin-4-amine [47] and phosphoaminophosphonic acid adenylate ester [48], with subsequent repairing and fixation of amide of side chains. Optimization protocols for minimizing variables were performed using the Powell conjugate gradient algorithm [49]; a dielectric constant value of 1.0, gradient convergence fixed to 0.005 kcal/mol, maximum number of iterations of 1000, and Kollman United/All-atoms force fields using AMBER charges. Followed by a format conversion into PDBQT file, necessary for Vina using AutoDock Tools.

A comparative structural analysis for the HER receptors was performed with SYBYL-X 2.0 by multiple sequence alignments, that arrange any given protein sequence into a rectangular array, in order to depict if residues in any given column are homologous, superposable or play a functional role (% of identity, % ID) and root mean square deviation (RMSD in Å), which depicts spatial similarity. These experiments were performed for whole proteins, as well as for the ligand binding site on each evaluated HER receptor.

#### 3.1.2. Preparation of NP Ligand Library (NatProd Collection)

The 800 natural compound structures, derived from animal, plant and microbial sources used during the in silico experiments, were obtained from the NatProd Collection database (http://www.msdiscovery.com/natprod.html), which embraces a diverse group of NPs, commercially available and carefully chosen according to literature reports that include: simple and complex stuctures of oxygen heterocycles, as well as, alkaloids, flavanoids, sterols, triterpenes, diterpenes, sesquiterpenes, benzophenones, chalcones, stilbenes, limonoids, quassinoids, chromones, coumarins; and the encompasses quinones, quinonemethides, benzofurans, benzopyrans, rotenoids, xanthones, carbohydrates, benztropolones, depsides, and depsidones (Additional information found in Supplementary Materials, Table S8). 3D structures of natural compounds were optimized using molecular mechanic methods with SYBYL-X 2.0 package, with a Tripos force field, Gasteiger-Marsili charges, and gradient convergence of 0.001 kcal/mol. All optimizations were performed by set of 1000 as the maximum number of iterations.

#### 3.1.3. Docking Calculations of NPs on HER Receptors

Docking calculations with AutoDock Vina were defined by establishing a cube at the geometric center of the co-crystalized ligand present on each selected, with dimensions 24 × 24 × 24 Å, employing a grid point spacing of 1.0 Å. The *x*, *y*, and *z* coordinates for the center grid boxes on 2ITW were −49.4540, 0.2830 and −19.5250, the coordinates used for 3PP0 were 35.8180, 44.1040 and −11.6860, for 3LMG were 11.9250, −29.2010, 45.8290 and for 2R4B, −14.5650, 17.3600, −19.5250. Three docking runs were performed for each ligand, and the pose with the highest Affinity absolute value (kcal/mol) was saved. Finally, the mean Affinity score for best poses was taken as the value of the binding Affinity for a particular complex [50,51].

Validation protocols by re-docking of co-crystallized ligand were performed to assure the performance of docking calculations using AutoDock Vina program. The 3D structures of compounds were obtained from each evaluated HER receptor: 1,2,3,4-tetrahydrostaurosporine (HER1, 2ITW); 2-{2-[4-({5-chloro-6-[3-(trifluoromethyl)phenoxy]pyridin-3-yl}amino)-5*H*-pyrrolo[3,2-*d*]pyrimidin-5-yl]ethoxy}ethanol (HER2, 3PP0); phosphoaminophosphonic acid adenylate ester (HER3, LMG); and *N*-{3-chloro-4-[(3-fluorobenzyl)oxy]phenyl}-6-ethylthieno[3,2-*d*]pyrimidin-4-amine (HER4, PDB:2R4B). Fifty docking runs were carried out for each ligand using the same parameters for evaluated natural compounds. Moreover, RMSD values were calculated for experimental (co-crystallized) versus theoretical poses (calculated by AutoDock Vina) [50].

### 3.2. Identification of Main Interaction of Selected NPs on HER Kinase Domain

Identification of interacting residues at distances lower than 4 Å was carried out using PyMol program (PyMOL Molecular Graphics System, Version 1.8 Schrödinger, LLC, New York, NY, USA), a compelling molecular visualization tool used to deliver and animate 3D structures. The molecular interaction nature of the residues that interact with selected NPs on the HER receptors binding site was performed using LigandScout 3.1 program (Inte-Ligand, Vienna, Austria), which utilizes simplified pharmacophores to detect the number and type of existing ligand-residue interactions on the protein active site for a particular protein-ligand complex. The most important HER-NP complexes were visualized with PyMol program depicting the binding mode for selected NPs.

### 3.3. Computational Prediction of ADME-TOX Properties Using FAF-Drugs3

The generation of the data was obtained by submitting the SDF (structure data file) file of the molecules as input, into the ADME-Tox Filtering Tool (http://fafdrugs3.mti.univ-paris-diderot.frl). During this process, the application of simple filters is made taking into account many physicochemical properties such as molecular weight, polar surface area, log P (P = octanol/water partition coefficient) or number of rotatable bonds and the existence of unfavorable chemical groups, toxic components and aggregators that may interfere in the calculations made in posterior in silico experiments. In addition to the identification of PAINS structures, considered embedded undesired chemical features also known as frequent hitters, promiscuous compounds and false positive inhibitors, that show biological activity by interfering with non-target organisms in an unselective manner [52]. These critical parameters are used to determine whether or not, the promissory NPs from the compound collection with the highest Affinity absolute values, have a greater probability of success as drug candidates, and therefore can be appropriately optimized. 

### 3.4. Molecular Docking Validation Using Reported Biological Data for HER Inhibitors

3D structures and biological data for a total of 113 known inhibitors were extracted from PubChem (https://pubchem.ncbi.nlm.nih.gov/) to validate the obtained results using molecular docking protocols, along with their binding affinities and total scores, which were calculated with AutoDock Vina following the same parameters described for the selected promissory natural compounds on their specific receptor. The biological information consisted of the half maximal inhibitory concentration (µM, IC_50_) values for HER1, HER2 and HER4 activity, as reported for these chemicals in PubChem BioAssay (PDB: 2ITW; AID: 439896, Inhibition of human recombinant HER1 expressed in Sf9 cells by liquid scintillation counting and AID: 66448, In vitro inhibition of HER1 autophosphorylation) (PDB: 3PP0; AID: 638068, Inhibition of Her-2 by time-resolved fluorescence assay, AID: 632228, Growth inhibition of human BT474 cells overexpressing HER2 gene after 5 days by particle analyzer and AID: 1062007, Inhibition of HER2 (unknown origin) by ADP-Glo assay) (PDB: 2R4B; AID: 260897, Inhibition of ErbB4 fusion protein expressed in baculovirus by ELISA and AID: 739684, Inhibition of HER4 cytoplasmic domain (unknown origin) expressed in baculovirus expression system incubated for 5 min prior to [gamma-32P]ATP addition measured after 10 min by scintillation counting analysis). A correlation analysis was made to determine relationships between the binding scores on HER Receptors 1, 2 and 4, generated by AutoDock Vina and experimental biological data (Log IC_50_).

## 4. Conclusions

The use of bioinformatics to identify possible protein inhibitors has great relevance for drug discovery. Obtained AutoDock Vina affinities (kcal/mol) for the kinase domain of HER receptors suggest that some natural compounds such as podototarin (−10.7), hecogenin acetate (−11.2), hesperidin (−11.5) and theaflavin (−10.7), known anticancer agents, have the potential to interact with these proteins, as their theoretical affinities are comparable with those calculated for known inhibitors of HER-tyrosine kinase receptors. This information is of special interest due the fact that their anticancer mechanisms are not completely understood. Natural inhibitors of HER receptors, identified by in silico screening of the NatProd Collection library, can therefore serve as scaffolds for designing new chemopreventive agents.

## Figures and Tables

**Table 1:** AutoDock Vina-calculated affinities (kcal/mol) for virtual hits compound as potential HER inhibitors.

Compound	HER1 (PDB: 2ITW)	HER2 (PDB: 3PP0)	HER3 (PDB: 3LMG)	HER4 (PDB: 2R4B)	Natural Source	Ref.
Podototarin	−10.7 ± 0.0	−9.5 ± 0.05	−9.0 ± 0.0	−8.8 ± 0.0	*Podocarpus* spp.	[26]
Hecogenin acetate	−10.1 ± 0.0	−11.2 ± 0.0	−10.2 ± 0.0	−10.6 ± 0.0	Genus Agave	[27]
Hesperidin	−8.4 ± 0.1	−10.5 ± 0.0	−11.5 ± 0.0	−9.4 ± 0.0	Citrus Fruits	[28]
Theaflavin	−9.0 ± 0.0	−10.8 ± 0.6	−10.5 ± 0.1	−10.7 ± 0.0	Black teas	[29]

The most negative affinity values for all protein structures are shown in bold. Ref.: References.

**Table 2:** ADME/TOX properties of the NPs proposed as inhibitors for HER receptors using FAF-Drugs3.

Natural Product	Molecular Descriptors ^a^											
	MW	Log P	Log D	tPSA	Rotable Bonds	Rigid Bonds	Flexibility	HBD	HBA	Sol (mg/L)	OB (VEBER)	OB (EGAN)
**Podotoarin**	570.89	13.13	12.18	40.46	3	32	0.09	2	2	5.5	Good	Good
**Hecogenin acetate**	472.66	5.40	5.11	61.83	2	32	0.06	0	5	1125.1	Good	Good
**Hersperidin**	610.56	−10.09	−0.32	234.29	7	30	0.19	8	15	45,708.2	Good	Good
**Theaflavin**	564.49	2.38	2.78	217.60	2	35	0.05	9	12	3922.9	Good	Good

^a^ MW: Molecular weight in Da; log P = Octanol-water partition coefficient; tPSA = topological polar surface area; HBA = Hydrogen bond atoms; HBD = Hydrogen bond donors; Sol = solubility; OB = Oral bioavailability.

## Supplementary Materials

Supplementary materials are available online.

## References

[1] Martinez-Useros J., Garcia-Foncillas J. (2015). Review: The challenge of blocking a wider family members of EGFR against head and neck squamous cell carcinomas. Oral Oncol..

[2] Tas F., Bilgin E., Karabulut S., Duranyildiz D. (2015). Clinical significance of serum epidermal growth factor receptor (EGFR) levels in patients with breast cancer. Cytokine.

[3] Marmor M.D., Skaria K.B., Yarden Y. (2004). EGFR INHIBITORS: Signal transduction and oncogenesis by ErbB/HER receptors. Int. J. Radiat. Oncol. Biol. Phys..

[4] RoskoskiRobert J. (2014). Invited Review: ErbB/HER protein-tyrosine kinases: Structures and small molecule inhibitors. Pharmacol. Res..

[5] Gonzales A.J., Fry D.W. (2005). G1 cell cycle arrest due to the inhibition of erbB family receptor tyrosine kinases does not require the retinoblastoma protein. Exp. Cell Res..

[6] Paul M.K., Mukhopadhyay A.K. (2004). Tyrosine kinase—Role and significance in Cancer. Int. J. Med. Sci..

[7] Navarro L., Gil-Benso R., Megías J., Muñoz-Hidalgo L., San-Miguel T., Callaghan R.C., González-Darder J.M., López-Ginés C., Cerdá-Nicolás M.J. (2015). Alteration of major vault protein in human glioblastoma and its relation with EGFR and PTEN status. Neuroscience.

[8] Mooso B.A., Vinall R.L., Mudryj M., Yap S.A., deVere White R.W., Ghosh P.M. (2015). Review Article: The Role of EGFR Family Inhibitors in Muscle Invasive Bladder Cancer: A Review of Clinical Data and Molecular Evidence. J. Urol..

[9] Song Z., Ge Y., Wang C., Huang S., Shu X., Liu K., Zhou Y., Ma X. (2016). Challenges and Perspectives on the Development of Small-Molecule EGFR Inhibitors against T790M-Mediated Resistance in Non-Small-Cell Lung Cancer. J. Med. Chem..

[10] Uribe P., Gonzalez S. (2011). Review: Epidermal growth factor receptor (EGFR) and squamous cell carcinoma of the skin: Molecular bases for EGFR-targeted therapy. Pathol. Res. Pract..

[11] Wang L., Yuan H., Li Y., Han Y. (2014). Review: The role of HER3 in gastric cancer. Biomed. Pharmacother..

[12] Mehta A., Tripathy D. (2014). Co-targeting estrogen receptor and HER2 pathways in breast cancer. Breast.

[13] Oh S., Ju J., Yang W., Lee K., Nam K., Shin I. (2015). EGFR negates the proliferative effect of oncogenic HER2 in MDA-MB-231 cells. Arch. Biochem. Biophys..

[14] Tai W., Mahato R., Cheng K. (2010). Review: The role of HER2 in cancer therapy and targeted drug delivery. J. Control. Release.

[15] Kol A., van Scheltinga A.G.T., Timmer-Bosscha H., Lamberts L.E., Bensch F., de Vries E.G.E., Schröder C.P. (2014). HER3, serious partner in crime: therapeutic approaches and potential biomarkers for effect of HER3-targeting. Pharmacol. Ther..

[16] Schneider M.R., Yarden Y. (2014). Structure and function of epigen, the last EGFR ligand. Semin. Cell Dev. Biol..

[17] Sebastian S., Settleman J., Reshkin S.J., Azzariti A., Bellizzi A., Paradiso A. (2006). The complexity of targeting EGFR signalling in cancer: From expression to turnover. Biochim. Biophys. Acta (BBA) Rev. Cancer.

[18] Mudjupa C., Abdelhamed S., Refaat A., Yokoyama S., Saiki I., Vajragupta O. (2015). Original Research Article: Lead compound bearing caffeic scaffold induces EGFR suppression in solid tumor cancer cells. J. Appl. Biomed..

[19] Kempf E., Lacroix L., Soria J.-C. (2015). First Reported Case of Unexpected Response to an Epidermal Growth Factor Receptor Tyrosine Kinase Inhibitor in the I744M Uncommon EGFR Mutation. Clin. Lung Cancer.

[20] Mehra R., Serebriiskii I.G., DunbrackRoland L.J., Robinson M.K., Burtness B., Golemis E.A. (2011). Protein-intrinsic and signaling network-based sources of resistance to EGFR- and ErbB family-targeted therapies in head and neck cancer. Drug Resist. Updates.

[21] Remon J., Morán T., Majem M., Reguart N., Dalmau E., Márquez-Medina D., Lianes P. (2014). Acquired resistance to epidermal growth factor receptor tyrosine kinase inhibitors in EGFR-mutant non-small cell lung cancer: A new era begins. Cancer Treat. Rev..

[22] Denis M.G., Vallée A., Théoleyre S. (2015). EGFR T790M resistance mutation in non small-cell lung carcinoma. Clin. Chim. Acta.

[23] Yim-Im W., Sawatdichaikul O., Semsri S., Horata N., Mokmak W., Tongsima S., Suksamrarn A., Choowongkomon K. (2014). Computational analyses of curcuminoid analogs against kinase domain of HER. BMC Bioinform..

[24] Trott O., Olson A.J. (2010). AutoDock Vina: Improving the speed and accuracy of docking with a new scoring function, efficient optimization and multithreading. J. Comput. Chem..

[25] Forli S., Huey R., Pique M.E., Sanner M.F., Goodsell D.S., Olson A.J. (2016). Computational protein-ligand docking and virtual drug screening with the AutoDock suite. Nat. Protoc..

[26] Cambie R.C., Madden R.J., Parnell J.C. (1971). Chemistry of the Podocarpaceae. XXVIII. Constituents of some Podocarpus and other species. Aust. J. Chem..

[27] Gama K.B., Quintans J.S.S., Antoniolli A.R., Quintans-Júnior L.J., Santana W.A., Branco A., Soares M.B.P., Villarreal C.F. (2013). Evidence for the involvement of descending pain-inhibitory mechanisms in the antinociceptive effect of hecogenin acetate. J. Nat. Prod..

[28] Devi K.P., Rajavel T., Nabavi S.F., Setzer W.N., Ahmadi A., Mansouri K., Nabavi S.M. (2015). Hesperidin: A promising anticancer agent from nature. Ind. Crop. Prod..

[29] Kong L., Qi X., Huang S., Chen S., Wu Y., Zhao L. (2015). Theaflavins inhibit pathogenic properties of *P. gingivalis* and MMPs production in *P. gingivalis*-stimulated human gingival fibroblasts. Arch. Oral Biol..

[30] Reddy P.J., Ray S., Sathe G.J., Gajbhiye A., Prasad T.S.K., Rapole S., Panda D., Srivastava S. (2015). A comprehensive proteomic analysis of totarol induced alterations in Bacillus subtilis by multipronged quantitative proteomics. J. Proteom..

[31] Gasparotto J., Somensi N., Kunzler A., Girardi C.S., de Bittencourt Pasquali M.A., Ramos V.M., Simoes-Pires A., Quintans-Junior L.J., Branco A., Moreira J.C.F. (2014). Hecogenin acetate inhibits reactive oxygen species production and induces cell cycle arrest and senescence in the A549 human lung cancer cell line. Anticancer Agents Med. Chem..

[32] Omar H.A., Mohamed W.R., Arafa E.-S.A., Shehata B.A., El Sherbiny G.A., Arab H.H., Elgendy A.N.A.M. (2016). Original research article: Hesperidin alleviates cisplatin-induced hepatotoxicity in rats without inhibiting its antitumor activity. Pharmacol. Rep..

[33] Bigoniya P., Singh K. (2014). Original article: Ulcer protective potential of standardized hesperidin, a citrus flavonoid isolated from Citrus sinensis. Rev. Bras. Farmacogn..

[34] Filho C.B., Del Fabbro L., de Gomes M.G., Goes A.T.R., Souza L.C., Boeira S.P., Jesse C.R. (2013). Kappa-opioid receptors mediate the antidepressant-like activity of hesperidin in the mouse forced swimming test. Eur. J. Pharmacol..

[35] Souza L.C., de Gomes M.G., Goes A.T.R., Del Fabbro L., Filho C.B., Boeira S.P., Jesse C.R. (2013). Evidence for the involvement of the serotonergic 5-HT1A receptors in the antidepressant-like effect caused by hesperidin in mice. Prog. Neuropsychopharmacol. Biol. Psychiatry.

[36] Siddiqi A., Hasan S.K., Nafees S., Rashid S., Saidullah B., Sultana S. (2015). Chemopreventive efficacy of hesperidin against chemically induced nephrotoxicity and renal carcinogenesis via amelioration of oxidative stress and modulation of multiple molecular pathways. Exp. Mol. Pathol..

[37] Zu M., Yang F., Zhou W., Liu A., Du G., Zheng L. (2012). In vitro anti-influenza virus and anti-inflammatory activities of theaflavin derivatives. Antivir. Res..

[38] Anandhan A., Tamilselvam K., Radhiga T., Rao S., Essa M.M., Manivasagam T. (2012). Theaflavin, a black tea polyphenol, protects nigral dopaminergic neurons against chronic MPTP/probenecid induced Parkinson’s disease. Brain Res..

[39] Vermeer M.A., Mulder T.P.J., Molhuizen H.O.F. (2008). Theaflavins from Black Tea, Especially Theaflavin-3-gallate, Reduce the Incorporation of Cholesterol into Mixed Micelles. J. Agric. Food Chem..

[40] Gao Y., Li W., Jia L., Li B., Chen Y.C., Tu Y. (2013). Enhancement of (−)-epigallocatechin-3-gallate and theaflavin-3–3′-digallate induced apoptosis by ascorbic acid in human lung adenocarcinoma SPC-A-1 cells and esophageal carcinoma Eca-109 cells via MAPK pathways. Biochem. Biophys. Res. Commun..

[41] De Oliveira A., Prince D., Lo C.-Y., Lee L.H., Chu T.-C. (2015). Antiviral activity of theaflavin digallate against herpes simplex virus type 1. Antivir. Res..

[42] Cabarcas-Montalvo M., Maldonado-Rojas W., Montes-Grajales D., Bertel-Sevilla A., Wagner-Döbler I., Sztajer H., Reck M., Flechas-Alarcon M., Ocazionez R., Olivero-Verbel J. (2016). Research paper: Discovery of antiviral molecules for dengue: In silico search and biological evaluation. Eur. J. Med. Chem..

[43] Jura N., Shan Y., Cao X., Shaw D.E., Kuriyan J. (2009). Structural Analysis of the Catalytically Inactive Kinase Domain of the Human EGF Receptor 3. Proc. Natl. Acad. Sci. USA.

[44] Wolber G., Langer T. (2005). LigandScout: 3-D pharmacophores derived from protein-bound ligands and their useas virtual screening filters. J. Chem. Inf. Model..

[45] Yun C.-H., Boggon T.J., Li Y., Woo M.S., Greulich H., Meyerson M., Eck M.J. (2007). Structures of Lung Cancer-Derived EGFR Mutants and Inhibitor Complexes: Mechanism of Activation and Insights into Differential Inhibitor Sensitivity. Cancer Cell.

[46] Aertgeerts K., Skene R., Yano J., Sang B.-C., Zou H., Snell G., Jennings A., Iwamoto K., Habuka N., Hirokawa A. (2011). Structural analysis of the mechanism of inhibition and allosteric activation of the kinase domain of HER2 protein. J. Biol. Chem..

[47] Wood E.R., Shewchuk L.M., Ellis B., Brignola P., Brashear R.L., Caferro T.R., Dickerson S.H., Dickson H.D., Donaldson K.H., Gaul M. (2008). 6-Ethynylthieno[3,2-*d*]- and 6-ethynylthieno[2,3-*d*]pyrimidin-4-anilines as tunable covalent modifiers of ErbB kinases. Proc. Natl. Acad. Sci. USA.

[48] Shi F., Telesco S.E., Liu Y., Radhakrishnan R., Lemmon M.A. (2010). ErbB3/HER3 intracellular domain is competent to bind ATP and catalyze autophosphorylation. Proc. Natl. Acad. Sci. USA.

[49] Powell M.J.D. (1977). Restart procedures for the conjugate gradient method. Math. Program..

[50] Maldonado-Rojas W., Olivero-Verbel J., Marrero-Ponce Y. (2015). Computational fishing of new DNA methyltransferase inhibitors from natural products. J. Mol. Graph. Model..

[51] Maldonado-Rojas W., Olivero-Verbel J. (2012). Food-related compounds that modulate expression of inducible nitric oxide synthase may act as its inhibitors. Molecules.

[52] Pouliot M., Jeanmart S. (2016). Pan Assay Interference Compounds (PAINS) and Other Promiscuous Compounds in Antifungal Research. J. Med. Chem..

