# Supplementary material for: Natural Products as Chemopreventive Agents by Potential Inhibition of the Kinase Domain in ErbB Receptors
Source: Molecules. 2017 Feb 17;22(2):308. doi: 10.3390/molecules22020308 (PMC6155853; doi:10.3390/molecules22020308)
Supplement: Supplementary file 1 [file molecules-22-00308-s001.pdf]

Supplementary Materials: Natural Products as Chemopreventive Agents by Potential Inhibition of the Kinase Domain in ErBb Receptors

Maria Olivero-Acosta, Wilson Maldonado-Rojas and Jesus Olivero-Verbel

Table S1. Protein characterization of human HER Receptor structures downloaded from PDB database.

Receptor Type	PDB Code	Name	Chain	residues	Ligand	Method	Resolution
HER 1	**2ITW**	Epidermal growth factor receptor	A	327	1,2,3,4-tetrahydrogen staurosporine	X-ray diffraction	2.88
HER 2	**3PP0**	Receptor tyrosine-protein kinase erbb-2	A, B	338	2-{2-[4-({5-chloro-6-[3-(trifluoromethyl)phenoxy]pyridin-3-yl}amino)-5h-pyrrolo[3,2-d]pyrimidin-5-yl]ethoxy}ethanol	X-ray diffraction	2.25
HER 3	**3LMG**	Receptor tyrosine-protein kinase erbb-3	A, B	344	Phosphoaminophosphonic acid-adenylate ester	X-ray diffraction	2.8
HER 4	**2R4B**	Receptor tyrosine-protein kinase erbb-4	A, B	321	*N*-{3-chloro-4-[(3-fluorobenzyl)oxy]phenyl}-6-ethylthieno[3,2-d]pyrimidin-4-amine	X-ray diffraction	2.4

Table S2. Results of Multiple Alignment of Sequence Identity (%ID) Performed by SYBYL X-2.0 for Four HER Receptors.

	Human Her			
**PDB CODE**	**2ITW**	**2R4B**	**3LMG**	**3PP0**
**2ITW** (HER1)	100.0	80.3	65.9	82.7
**2R4B** (HER4)	80.3	100	71.7	80.9
**3LMG** (HER3)	65.9	71.7	100	67.4
**3PP0** (HER2)	82.7	80.9	67.4	100

Table S3. Multiple alignment of spatial coordinates for HER receptor pairs (by RMSD) using SYBYL X-2.0.

	Human Her			
**PDB CODE**	**2ITW**	**2R4B**	**3LMG**	**3PP0**
**2ITW** (HER1)	0	4.378	4.162	5.682
**2R4B** (HER4)	4.378	0	2.958	3.31
**3LMG** (HER3)	4.162	2.958	0	3.656
**3PP0** (HER2)	5.682	3.31	3.656	0

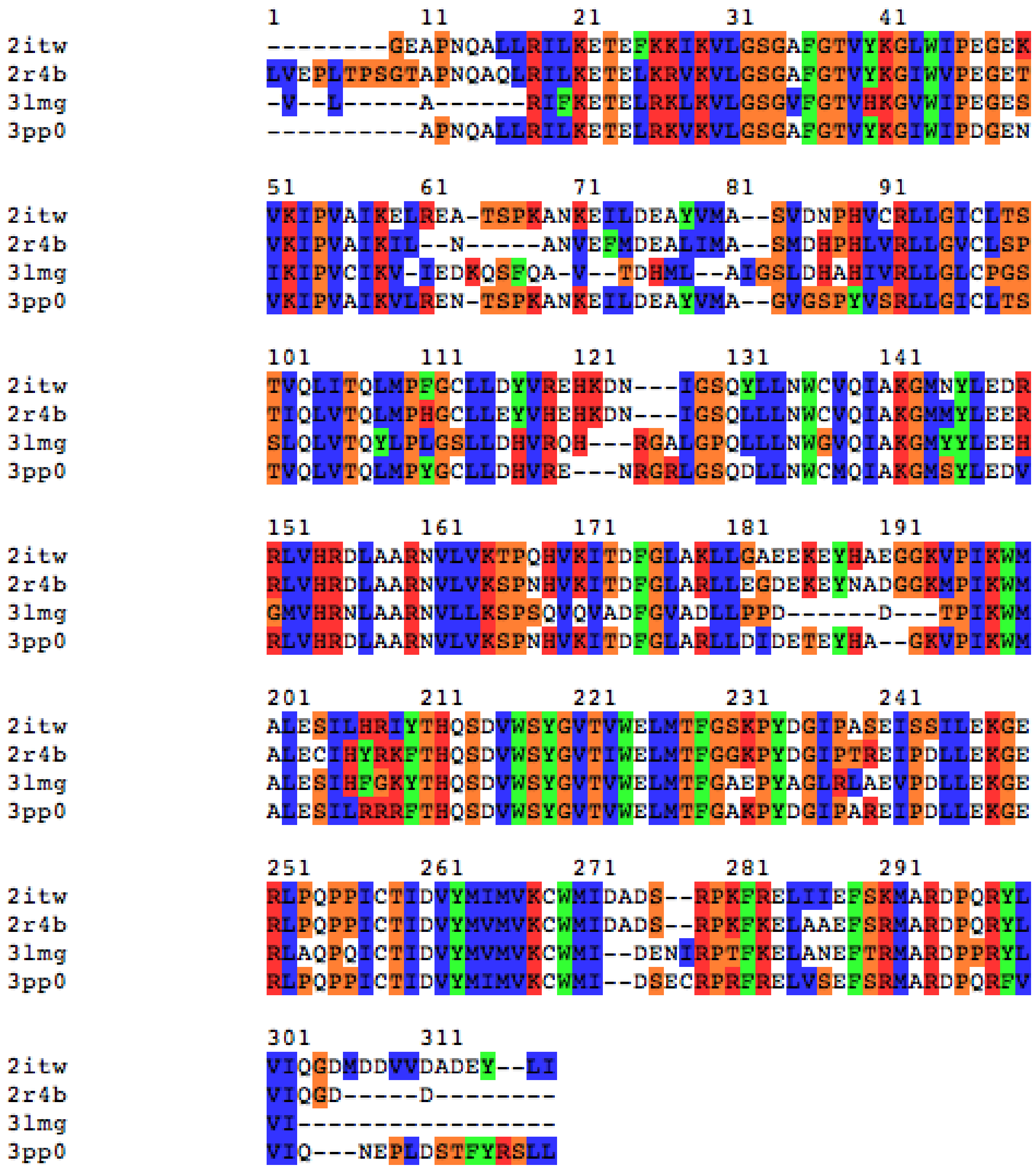

Figure S1. Multiple alignments of amino acid sequences from evaluated HER structures using Sybyl X-2.0 program.

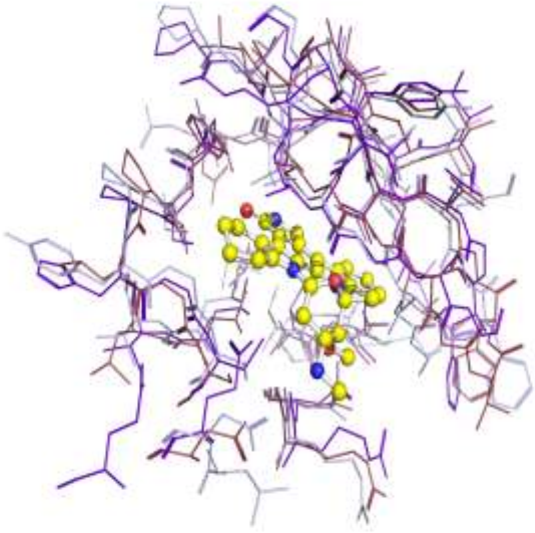

a. **Structural superposition for HER kinase domain binding sites (HER1, HER2, HER3 and HER4)**

b. Multiple sequence alignment

```
            1         11        21        31        41
2itw        KV-GSGAFGTVYK-P-AIKEMCLTQLM-FGC-DRNLVKT-D
2r4b        KVLGSG-FGTVYKGPVAIKIVT---LMPHGCEERNLVTDL-
3lmg        KVLGSGVFGTVHK--VCIKVVLVTQYLPLGS-DRNLLADV-
3pp0        KVLGSGAFGTVYKGPVAIKVLSLTQLMPYGCLDRNLVTDFG

Background Color: ClustalX
Joy Annotation:
 alpha helix                          red          x
 beta strand                          blue         x
 3 - 10 helix                         maroon       x
```

c. % Sequence identity and RMSD (Å)

Pairwise Sequence Identity:				
Protein	1	2	3	4
1 2itw	100.0	76.7	57.6	80.0
2 2r4b	76.7	100	63.6	80.6
3 3lmg	57.6	63.6	100.0	64.9
4 3pp0	80.0	80.6	64.9	100.0
**Pairwise C-Alpha RMSD:**				
	1	2	3	4
1 2itw	0.00	4.10	4.18	4.41
2 2r4b	4.10	0.00	1.81	3.60
3 3lmg	4.18	1.81	0.00	3.72
4 3pp0	4.41	3.60	3.72	0.00

Figure S2. Structural superposition of four binding sites from HER kinase domain (1–4) (**a**); with the visualization of multiple sequence alignment (Clustal X) (**b**); and determination of percentage sequence and RMSD (**c**) matrix.

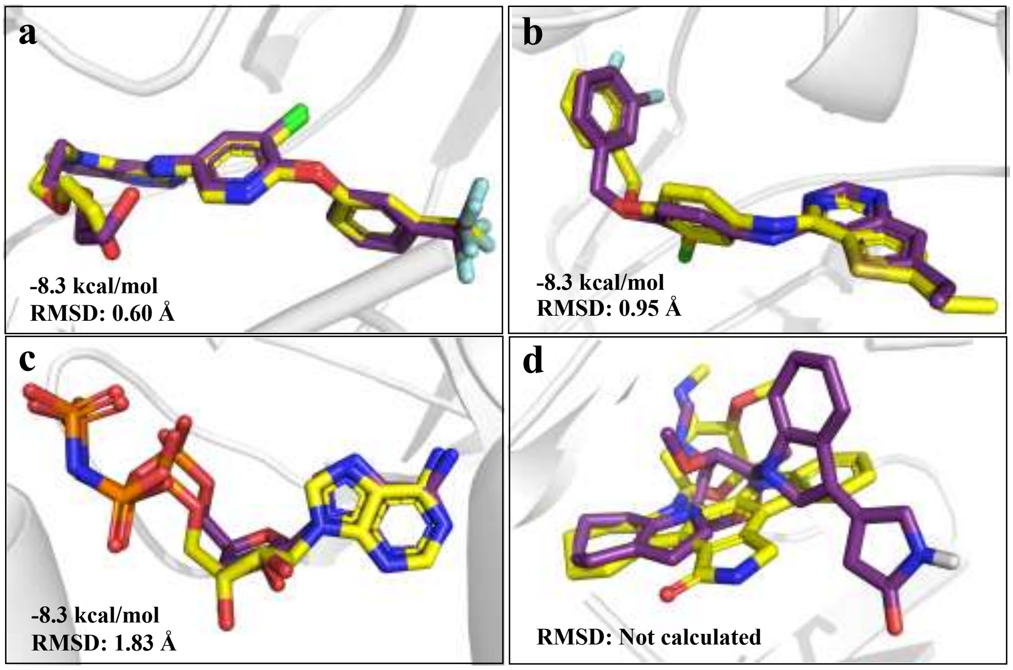

Figure S3. Molecular superposition between predicted with AutoDock Vina (yellow) and co-crystallized pose (purple) co-crystallized ligand for HER2 (3PP0) (**a**); HER4 (2R4B) (**b**); HER3 (3LMG) (**c**); and HER1 (2ITW). The RMSD values for 2ITW were not calculated due to fragmentation of their co-crystallized ligand in posterior docking procedures.

Table S4. AutoDock Vina Affinity scores for ligands on HER1 and their half maximal inhibitory concentrations (IC_{50}).

No.	CID	Mean AV Affinity (Kcal/mol)	IC_{50} (μM)	Log IC_{50}
1.	45483677	−8.7 ± 0.05	0.019	−1.721
2.	45483696	−8.7 ± 0.05	0.033	−1.481
3.	45483687	−8.6 ± 0.05	0.030	−1.523
4.	45483674	−8.6 ± 0.0	0.023	−1.638
5.	45483694	−8.6 ± 0.14	0.045	−1.347
6.	45483675	−8.6 ± 0.05	0.023	−1.638
7.	45483689	−8.5 ± 0.0	0.038	−1.420
8.	10437018	−8.3 ± 0.08	0.032	−1.495
9.	45483685	−8.2 ± 0.08	0.044	−1.357
10.	45483693	−8.1 ± 0.05	0.038	−1.420
11.	5328762	−8.1 ± 0.0	15.000	1.176
12.	1838043	−8.1 ± 0.05	12.000	1.079
13.	45483699	−8.0 ± 0.12	0.111	−0.955
14.	45483691	−8.0 ± 0.0	0.040	−1.398
15.	45483695	−8.0 ± 0.14	0.021	−1.678
16.	5328763	−7.8 ± 0.19	14.000	1.146
17.	5328770	−7.8 ± 0.05	0.700	−0.155
18.	5328767	−7.7 ± 0.0	24.000	1.380
19.	5328765	−7.7 ± 0.19	11.000	1.041
20.	5328779	−7.7 ± 0.05	0.100	−1.000
21.	5328760	−7.6 ± 0.19	5.000	0.699
22.	5328781	−7.5 ± 0.05	10.300	1.013
23.	5328764	−7.4 ± 0.0	6.600	0.820
24.	5328774	−7.4 ± 0.0	2.100	0.322
25.	5328775	−7.4 ± 0.0	5.000	0.699
26.	5328776	−7.4 ± 0.05	49.000	1.690
27.	5328771	−7.3 ± 0.0	1.250	0.097
28.	5328773	−7.2 ± 0.0	0.625	−0.204
29.	5353388	−7.2 ± 0.0	2.500	0.398
30.	2385464	−7.0 ± 0.0	5.900	0.771
31.	5328766	−7.0 ± 0.0	31.000	1.491
32.	5328768	−6.0 ± 0.0	4.500	0.653

Table S5. AutoDock Vina Affinity scores for ligands on HER2and their half maximal inhibitory concentrations (IC_{50}).

No	CID	Binding Affinity	IC_{50}(uM)	Log IC_{50}
1.	56835161	−11.5 ± 0.0	0.062	−1.208
2.	11679357	−11.1 ± 0.1	0.009	−2.046
3.	57392589	−11.0 ± 0.0	0.140	−0.854
4.	57390776	−11.0 ± 0.1	0.041	−1.387
5.	11955109	−10.7 ± 0.0	0.018	−1.745
6.	57392590	−10.7 ± 0.2	0.110	−0.959
7.	57396070	−10.7 ± 0.1	0.130	−0.886
8.	57397871	−10.6 ± 0.0	0.220	−0.658
9.	57400426	−10.6 ± 0.0	0.073	−1.137
10.	57401286	−10.5 ± 0.0	0.086	−1.066
11.	57390777	−10.5 ± 0.1	0.030	−1.523
12.	57392591	−10.4 ± 0.1	0.120	−0.921
13.	11620908	−10.2 ± 0.0	0.017	−1.770
14.	56849848	−10.2 ± 0.0	0.050	−1.301
15.	57397872	−10.2 ± 0.0	0.180	−0.745
16.	57392588	−10.1 ± 0.1	0.041	−1.387
17.	56849846	−10.0 ± 0.1	0.124	−0.907

18.	57401285	−10.0 ± 0.1	0.750	−0.125
19.	56849849	−9.9 ± 0.0	0.061	−1.215
20.	56849562	−9.8 ± 0.0	1.884	0.275
21.	56849847	−9.8 ± 0.1	0.210	−0.678
22.	56849560	−9.7 ± 0.0	0.692	−0.160
23.	56851591	−9.6 ± 0.0	1.063	0.027
24.	56851589	−9.5 ± 0.0	0.875	−0.058
25.	56851594	−9.5 ± 0.2	0.650	−0.187
26.	56851288	−9.5 ± 0.1	1.143	0.058
27.	56851053	−9.4 ± 0.1	1.076	0.032
28.	123631	−9.4 ± 0.0	0.240	−0.620
29.	56851052	−9.4 ± 0.0	1.378	0.139
30.	56851588	−9.3 ± 0.0	2.328	0.367
31.	56851590	−9.3 ± 0.1	2.543	0.405
32.	56851166	−9.3 ± 0.1	0.069	−1.161
33.	56851432	−9.3 ± 0.1	0.787	−0.104
34.	56850930	−9.2 ± 0.1	0.601	−0.221
35.	11684629	−9.2 ± 0.1	0.027	−1.569
36.	56849702	−9.2 ± 0.0	0.582	−0.235
37.	56849701	−9.1 ± 0.0	0.212	−0.674
38.	16736274	−9.0 ± 0.1	2.170	0.336
39.	56850806	−9.0 ± 0.2	0.406	−0.391
40.	56851051	−9.0 ± 0.3	0.699	−0.156
41.	56849558	−9.0 ± 0.0	0.184	−0.735
42.	56851431	−8.9 ± 0.1	0.956	−0.020
43.	56851592	−8.9 ± 0.0	0.762	−0.118
44.	56851428	−8.9 ± 0.1	1.267	0.103
45.	56849555	−8.8 ± 0.0	1.160	0.064
46.	56851429	−8.8 ± 0.0	1.413	0.150
47.	56851593	−8.8 ± 0.0	1.216	0.085
48.	56851430	−8.8 ± 0.1	0.459	−0.338
49.	56851427	−8.7 ± 0.1	0.737	−0.133
50.	56849704	−8.7 ± 0.0	0.116	−0.936
51.	56849556	−8.7 ± 0.5	2.049	0.312
52.	56851290	−8.6 ± 0.1	0.869	−0.061
53.	56851433	−8.6 ± 0.1	4.473	0.651
54.	56849563	−8.5 ± 0.1	0.097	−1.013
55.	56849703	−8.4 ± 0.0	0.054	−1.268
56.	176870	−7.9 ± 0.1	0.760	−0.119
57.	10205	−7.4 ± 0.0	30.800	1.489
Co-crystallized ligand	2-{2-[4-({5-chloro-6-[3-(trifluoromethyl)phenoxy]pyridin-3-yl}amino)-5*H*-pyrrolo[3,2-d]pyrimidin-5-yl]ethoxy}etanol (SYR127063)	−10.9 ± 0.0	0.011	−1.959

Table S6. AutoDock Vina Affinity scores for ligands on HER4 and their half maximal inhibitory concentrations (IC_{50}).

No.	CID	Binding Affinity	IC_{50}	$LogIC_{50}$
1.	11503073	−10.1 ± 0.05	0.229	−0.640
2.	11604864	−10.0 ± 0.09	0.0018	−2.745
3.	2776	−10.0± 0.05	0.012	−1.921
4.	11632391	−10.0 ± 0.05	0.0024	−2.620
5.	11524106	−9.9 ± 0.0	0.0071	−2.149
6.	11539461	−9.8 ± 0.0	0.0062	−2.208
7.	9907221	−9.8 ± 0.0	0.0017	−2.770
8.	11711590	−9.7 ± 0.05	0.644	−0.191
9.	11590557	−9.6 ± 0.1	0.0043	−2.367
10.	11595844	−9.6 ± 0.0	0.0073	−2.137
11.	11568720	−9.6 ± 0.1	0.001	−3.000
12.	11713043	−9.6 ± 0.1	0.001	−3.000
13.	11566580	−9.6 ± 0.1	0.0005	−3.301
14.	11553647	−9.5 ± 0.0	0.31	−0.509
15.	18176200	−9.4 ± 0.1	0.013	−1.886
16.	11719423	−9.4 ± 0.0	0.011	−1.959
17.	156413	−9.3 ± 0.0	0.031	−1.509
18.	11518389	−9.2 ± 0.1	0.0012	−2.921
19.	11613187	−9.2 ± 0.1	0.0008	−3.097
20.	11532242	−9.2 ± 0.1	0.016	−1.796
21.	11496207	−9.1± 0.0	0.538	−0.269
22.	71583657	−8.9 ± 0.1	0.24	−0.620
23.	11518637	−8.4 ± 0.0	0.624	−0.205
24.	11634425	−8.2 ± 0.2	0.073	−1.137
Co-crystallized ligand	*N*-{3-chloro-4-[(3-fluorobenzyl)oxy]phenyl}-6-ethylthieno[3,2-d]pyrimidin-4-amine	−10.7 ± 0.0	0.060	−1.222

Table S7. 800 NPs from NatProd Collection, MicroSource Discovery Systems.

#	SYBYL-CODE	ID	MOLECULE NAME	Formula	MolWt	cas#	Class	Bioactivity	Source
1	538988232	01505672	VINCRISTINE SULFATE	C46H58N4O14S	923.06	2068-78-2	alkaloid	antineoplastic	Vinca rosea; 37231, NSC-67574
2	538988131	01505464	THIAMINE	C12H18Cl2N4OS	337.27	67-03-8	alkaloid	vitamin B1, enzyme cofactor	rice husks, wheat germ, yeast
3	538988127	01505453	PYRIDOXINE	C8H11NO3	169.18	65-23-6	alkaloid	vitamin B6, enzyme cofactor	rice husks, wheat germ, yeast
4	1206009	01500821	BICUCULLINE (+)	C20H17NO6	367.36	485-49-4	alkaloid	GABAa antagonist	*Dicentra cucullaria, Corydalis* spp.
5	538985342	01503922	TRYPTAMINE	C10H12N2	160.22	61-54-1	alkaloid	psychotropic	*Acacia* spp., *Lens esculenta*, *Prosopsis juliflora* and fungi *Ponaeolus foenisicii* and *Coprinus micaceus*
6	1073	01500655	ACONITINE	C34H47NO11	645.75	302-27-2	alkaloid	anesthetic (gastric), antipyretic, and cardiotoxin	*Aconitum*spp.
7	538987949	01505002	LAPPACONITINE	C32H44N2O8	584.72	32854-75-4	alkaloid	analgesic, antiarrhythmic	*Aconitum* spp. and *Delphinium cashmirianum*
8	538986699	00300010	AGELASINE (stereochemistry of diterpene unknown)	C26H40ClN5	458.10		alkaloid	cytotoxic, antineoplastic	*Agelas dispar*
9	538977864	00350011	AJMALINE DIACETATE	C24H30N2O4	410.52	19775-56-5	alkaloid		ajmaline derivative
10	538985919	00100279	LYCORINE	C16H17NO4	287.32	476-28-8	alkaloid		*Amarylidaceae* spp.
11	538977955	01503982	AGMATINE SULFATE	C5H16N4O4S	228.27	2482-00-0	alkaloid	NMDA blocker, alpha-2 adrenergic agonist; NO synthase inhibitor	*Ambrosia entemisifolia*
12	538986031	01504155	APHYLLIC ACID	C15H26N2O2	266.39	642-67-1	alkaloid		*Anabasis aphylla*
13	538986602	01505240	LUPANYL ACID HYDROCHLORIDE	C14H25ClN2O2	288.82		alkaloid		*Anabasis aphylla*
14	538986032	01504156	ANABASAMINE HYDROCHLORIDE	C16H20ClN3	289.81	20410-87-1(base)	alkaloid		*Anabasis aphylla*
15	538978159	00310004	ANABASINE HYDROCHLORIDE	C10H15ClN2	198.70	13078-04-1 (anabasine)	alkaloid	insecticide	*Anabasis aphylla & Nicotiana* spp.
16	538978111	01504021	LUPININE	C10H19NO	169.27	486-70-4	alkaloid	antifeedant, antiinflammatory, oxytoxic	*Anabasis aphylla, Lupinus* spp.

17	538978113	01504023	THERMOPSINE PERCHLORATE	C15H21ClN2O5	344.80	486-90-8(base)	alkaloid		*Anabasis aphylla, Thermopsis* spp.
18	1206020	01500866	HARMANE	C12H10N2	182.23	486-84-0	alkaloid	intercalating agent, sedative	*Arariba rubra.*
19	1201788	01500706	ARCAINE SULFATE	C6H18N6O4S	270.31	36587-93-6	alkaloid		*Arca noae*
20	538986247	01502126	GUVACINE HYDROCHLORIDE	C6H10ClNO2	163.61	498-96-4	alkaloid	muscarinic agonist, GABA uptake inhibitor	*Areca catechu*
21	538986722	01500878	TROPINE	C8H15NO	141.21	120-29-6	alkaloid		*Atropa belladonna, Hyoscyamus niger, Datura* spp..
22	1207072	01500876	CORYNANTHINE	C21H26N2O3	354.45	123333-62-0	alkaloid		bark of *Pseudocinchona africana Chev.*
23	1210806	01500811	BERBERINE CHLORIDE	C20H18ClNO4	371.82	633-65-8, 2086-83-1	alkaloid	antiarrhythmic, alpha2 agonist, cholinesterase, anticonvulsant, antiinflammatory, antibacterial, antifungal, antitrypanosomal, antineoplastic, immunostimulant; LD50(mouse) 329 mg/kg po	*Berberis and Mahonia* spp.
24	538978123	01501019	BERBAMINE HYDROCHLORIDE	C37H42Cl2N2O6	681.66	478-61-5 (berbamine)	alkaloid	antihypertensive, skeletal muscle relaxant	*Berberis* spp.
25	39	01500680	ARECOLINE HYDROBROMIDE	C8H14BrNO2	236.11	300-08-3, 63-75-2 [arecoline]	alkaloid	anthelmintic (Cestodes), hypotensive, cathartic	betel nuts (*Arica catechu*)
26	1207066	01500873	PIPERINE	C17H19NO3	285.35	94-62-2	alkaloid	analeptic, antibacterial	black pepper (*Piper nigrum L.)*
27	538986749	01505320	INDOLE-3-CARBINOL	C9H9NO	147.18	700-06-1	alkaloid	antineoplastic	*Brassica* spp.
28	462	01500649	THEOBROMINE	C7H8N4O2	180.17	83-67-0	alkaloid	diuretic, bronchodilator, cardiotonic	*Camelia, Theobroma, Cola* spp.
29	538985670	01502232	CAMPTOTHECIN	C20H16N2O4	348.36	7689-03-4	alkaloid	antineoplastic	*Camptotheca acuminata*
30	538986018	01504123	10-HYDROXYCAMPTO THECIN	C20H16N2O5	364.36	19685-09-7	alkaloid	antineoplastic	*Camptotheca acuminata*
31	538986436	00501332	PHENACYLAMINE	C8H10ClNO	171.63		alkaloid		*Castanopsis cuspidata,*

			HYDROCHLORIDE						*Vitis* spp.
32	1207078	01500826	CEPHALOTAXINE	C18H21NO4	315.37	24316-19-6	alkaloid		*Cephalotaxus drupacea and C. fortunii*
33	1206012	01500824	CHELIDONINE (+)	C20H19NO5	353.38	476-32-4	alkaloid	antineoplastic, smooth muscle relaxant, hypotensive, CNS depressant	*Chelidonium majus, Linn. (celandine)*
34	1207065	01500871	NORHARMAN	C11H8N2	168.20	244-63-3	alkaloid	plant growth inhibitor; mutagen	*Chrysophyllum and Nocardia* spp.
35	1200719	01500657	HYDROQUINIDINE	C20H26N2O2	326.44	1435-55-8	alkaloid	antiarrhythmic, antimalarial	Cinchona bark
36	1210789	01500839	CINCHONIDINE	C19H22N2O	294.40	485-71-2	alkaloid	antimalarial	*Cinchona* spp.
37	1210790	01500841	CINCHONINE	C19H22N2O	294.40	5949-16-6, 118-10-5 [cinchonine]	alkaloid	antimalarial	*Cinchona* spp.
38	538978120	01800067	COLCHICEINE	C21H23NO6	385.42	477-27-0	alkaloid	antimitotic	*Colchicum autumnale*
39	538978119	01500205	COLCHICINE	C22H25NO6	399.45	64-86-8	alkaloid	antimitotic, antigout agent	*Colchicum autumnale*
40	538976408	01502237	HARMOL HYDROCHLORIDE	C12H11ClN2O	234.69	40580-83-4	alkaloid	MAO inhibitor	common plant alkaloid
41	1210708	01500853	CHONDROSINE	C12H21NO11	355.30		alkaloid		component of chondroitin sulfate; mammalian amniotic fluid and placenta
42	143	01504178	TETRAHYDROPALMA TINE	C21H25NO4	355.44	3520-14-7	alkaloid	analgesic, hypnotic, papaverine-like	*Corydalis* spp.
43	538977860	01500663	YOHIMBINE HYDROCHLORIDE	C21H27ClN2O3	390.91	65-19-0	alkaloid	alpha adrenergic blocker, mydriatic, antidepressant	*Corynanthe* spp.
44	1201068	00100525	CRINAMINE	C17H19NO4	301.35	639-41-8	alkaloid	antihypertensive, respiratory depressant, antineoplastic	Crineum species
45	1200999	00100530	AMBELLINE	C18H21NO5	331.37	3660-62-6	alkaloid	immune stimulant, cytotoxic	*Crinium glaucum, Bruswigia rosea, Amaryllis belladonna*
46	538980230	01502252	MONOCROTALINE	C16H23NO6	325.36	315-22-0	alkaloid	antineoplastic, insect sterilant	*Crotalaria* spp.
47	538986149	01504205	DELTALINE	C24H37NO7	451.56	6836-11-9	alkaloid	antiarrhythmic	*Delphinium* spp.
48	538977878	00100599	2-METHYL GRAMINE	C12H16N2	188.27		alkaloid		derivative
49	1201558	00100563	OXONITINE	C33H43NO12	645.71		alkaloid		derivative of aconitine

50	538988023	01505169	DESOXYPEGANINE HYDROCHLORIDE	C11H13ClN2	208.69	61939-05-7	alkaloid	acetylcholinesterase inhibitor, antiParkinsonism	derivative of *peganine Peganum* spp.
51	538977073	02300324	PLECTOCOMINE METHYL ETHER	C12H14N2O	202.26		alkaloid	MAO inhibitor	derivative *Plectocomiopis* spp.
52	538977643	00310038	SCOPOLINE	C8H13NO2	155.20		alkaloid		dervative of scopolamine
53	1206016	01500860	S-ISOCORYDINE (+)	C20H23NO4	341.41	475-67-2	alkaloid	sedative, cholinergic	Dicentra canadensis, Artabotyris suaveoleus and Cordyalis species
54	1201148	00100541	DICTAMNINE	C12H9NO2	199.21	484-29-7	alkaloid		*Dictamnus albus* and other Rutaceae
55	1207079	01500939	NORELEAGNINE	C11H12N2	172.23		alkaloid		*Elaenus* spp.
56	538978110	01504020	EVOXINE	C18H21NO6	347.37	522-11-2	alkaloid		*Evodia xanthoxyloides*
57	1210907	01501202	GALANTHAMINE HYDROBROMIDE	C17H22BrNO3	368.27	357-70-0, 1953-04-4 [hydrobromide]	alkaloid	anticholinesterase, analgesic, antiAlzheimer	Galanthus, Narcissus and other Lillaceae
58	538977967	01503990	CONESSINE	C24H40N2	356.60	5913-82-6, 546-06-5 [conessine]	alkaloid		Holarrhena spp.
59	538977556	00300537	XANTHOPTERIN	C6H5N5O2	179.14	119-44-8	alkaloid	cell proliferation inhibitor	human urine, butterfly wing pigment
60	538985298	01501009	1R,9S-HYDRASTINE	C21H21NO6	383.40	118-08-1	alkaloid	uterine hemostatic, antiseptic	*Hydrastis canadensis*
61	1206023	01500872	PALMATINE CHLORIDE	C21H22ClNO4	387.87	10605-02-4	alkaloid	antibacterial, antimalarial, uterine contractant	*Jateorhiza palmata* (Calumba root)
62	1105	01502020	FOLIC ACID	C19H19N7O6	441.41	59-30-3	alkaloid	hematopoietic vitamin	liver, kidney, green plants and fungi
63	522	01500758	LOBELINE HYDROCHLORIDE	C22H28ClNO2	373.93	90-69-7	alkaloid	antiasthmatic, respiratory stimulant	*Lobelia* spp.
64	538986204	00100520	LUNARINE	C25H31N3O4	437.54	24185-51-1	alkaloid		*Lunaria* spp.
65	538986647	01505242	LUPANINE PERCHLORATE	C15H25ClN2O5	348.83	550-90-3 (base)	alkaloid		*Lupinus and Cystisus* spp.
66	538986644	00300548	SPARTEINE SULFATE	C15H28N2O4S	332.47	6160-12-9, 299-39-8 [anhydrous], 90-39-1 [sparteine]	alkaloid	oxytocic	*Lupinus* spp. *and other Leguminosae*
67	538986629	01505255	HUPERZINE A	C15H18N2O	242.32	102518-79-6	alkaloid	anticholinesterase, cognition enhancer	*Lycopodium* spp.

68	538977692	00100301	TOMATIDINE HYDROCHLORIDE	C27H46ClNO2	452.13	69004-03-1	alkaloid		*Lycospercion and Solanum* spp.
69	538986185	02300329	1-METHYLXANTHINE	C6H6N4O2	166.14	6136-37-4	alkaloid	diuretic, adenosine antagonist	major component of human urine
70	14	01500944	CARNOSINE	C9H14N4O3	226.24	305-84-0	alkaloid		mammalian skeletal muscle
71	538977738	01600300	CREATININE	C4H7N3O	113.12	60-27-5	alkaloid	metabolic enhancer	metabolite in muscle and urine; renal metabolite
72	538985634	01500208	COTININE	C10H12N2O	176.22	5695-98-7, 486-56-6 [cotinine]	alkaloid	antidepressant	*Nicotiana tabacum*
73	538977945	01503431	CYTIDINE	C9H13N3O5	243.22	65-46-3	alkaloid		nucleoside
74	1204212	00100537	SOLASODINE	C27H43NO2	413.65	126-17-0	alkaloid	antineoplastic, antiinflammatory	numerous *Solanum* spp.
75	1201072	00100595	COTARNINE CHLORIDE	C12H14ClNO3	255.70	10018-19-6, 82-54-2 [cotarnine]	alkaloid	vasoconstrictor	*Papaver pseudo-orientale*
76	538985902	01501014	HYDROCOTARNINE HYDROBROMIDE	C12H16BrNO3	302.17	550-10-7	alkaloid	vasoconstrictor	*Papaver somniferum*
77	538977938	01500754	XANTHURENIC ACID	C10H7NO4	205.17	59-00-7	alkaloid	caspase activator, guanylyl cyclase stimulant	pathological metabolite of tryptophan and kynurenine
78	1206021	01500867	HARMINE	C13H12N2O	212.25	442-51-3	alkaloid	antiparkinsonian, CNS stimulant	*Peganium harmala*
79	538976359	01500864	HARMALINE	C13H14N2O	214.27	304-21-2	alkaloid	CNS stimulant, antiparkinsonian agent	*Peganium harmala*
80	1206019	01500865	HARMALOL HYDROCHLORIDE	C12H13ClN2O	236.70	6028-07-5	alkaloid	anthelmintic, narcotic agent	*Peganium harmala*
81	1206017	01500862	BOLDINE	C19H21NO4	327.38	476-70-0	alkaloid		*Peumus boldus*
82	538977582	00200191	METHYL COCLAURINE	C18H21NO3	299.37		alkaloid		*Phylica rogersii*
83	296	01500690	MELATONIN	C13H16N2O2	232.28	73-31-4	alkaloid	sleep induction, modifies circadian rhythm	pineal gland
84	538987990	01505135	PIPLARTINE	C17H19NO5	317.34	20069-09-4	alkaloid	anti-asthma, antibronchitis	*Piper* spp.
85	538977519	00300553	PELLETIERINE HYDROCHLORIDE	C8H16ClNO	177.68		alkaloid		*Punica granatum*
86	538976366	01502227	CADAVERINE TARTRATE	C7H16N2O6	224.22	462-94-2(base)	alkaloid		putrifaction of lysine; sectrtion of the fox Vulpes vulpes

87	425	01500526	RESERPINE	C33H40N2O9	608.69	50-55-5	alkaloid	antihypertensive	Rauwolfia serpentina
88	1200504	01500656	AJMALINE	C20H26N2O2	326.44	4360-17-7	alkaloid	antiarrhythmic (Class Ia): inhibits glucose uptake by mitochondria, & PAF blocker	*Rauwolfia* spp., *Melodinus balansae, Tonduzia longifolia*
89	437	01503639	RAUWOLSCINE HYDROCHLORIDE	C21H27ClN2O3	390.91		alkaloid	alpha2 adrenergic antagonist	*Rauwolfia, Aspidosperma and Vinca* spp.
90	350	01500639	OCTOPAMINE HYDROCHLORIDE	C8H12ClNO2	189.64	104-14-3	alkaloid	adrenergic agonist	salivary glands of *Octopus vulgaris*; also *Capsicum frutescens & Cyperus* spp.
91	538978115	01504025	SALSOLINE	C11H15NO2	193.25	89-31-6	alkaloid	antihypertensive, antihistamine	*Salsola richteri*
92	538978112	01504022	SALSOLIDINE	C12H17NO2	207.27	493-48-1	alkaloid	antihypertensive	*Salsola Richteri*
93	538977640	00310035	SANGUINARINE SULFATE	C20H15NO8S	429.41	5578-73-4	alkaloid	antineoplastic, antiplaque agent	*Sanguinaria canadensis*
94	1201027	00300047	ANISODAMINE	C17H23NO4	305.38	17659-49-3	alkaloid	anticholinergic, antispasmodic	*Scopolia tanguticus*
95	538986752	01505334	SECURININE	C13H15NO2	217.27	5610-40-2	alkaloid	GABAA receptor blocker, CNS stimulant	*Securinega* spp. *and Phyllanthus discoides*
96	1200695	01500861	CORALYNE CHLORIDE	C22H22ClNO4	399.88	38989-38-7	alkaloid	cytostatic, intercalating agent	semisynthetic
97	538986741	01505322	CEPHARANTHINE	C37H38N2O6	606.73	481-49-2	alkaloid	antineoplastic, hepatoprotectant, radiopropective	Stephania spp.
98	78	01500624	CARNITINE (dl) HYDROCHLORIDE	C7H16ClNO3	197.66	461-06-3	alkaloid	antihyperlipoproteinemic, gastric/pancreatic secretion stimulant	striated muscle, liver; also in Pisum sativum
99	442	01500651	STRYCHNINE	C21H22N2O2	334.42	57-24-9	alkaloid	central stimulant	*Strychnos nux-vomica and other Strychnos* spp.
100	1206010	01500822	BRUCINE	C23H26N2O4	394.47	4845-99-2, 357-57-3 [brucine]	alkaloid	central stimulant	*Strychnos nux-vomica*
101	538978116	01504027	CYTISINE	C11H14N2O	190.25	485-35-8	alkaloid	antiinflammatory, respiratory stimulant	*Thermopsis lanceolata*
102	1207071	01500880	TRIGONELLINE	C7H7NO2	137.14	535-83-1	alkaloid	antihyperglycemic	*Trigonella foenumgraecum* and in coffee beans

103	1200657	01500272	EMETINE	C29H42Cl2N2O4	553.58	316-42-7, 483-18-1 [emetine]	alkaloid	inhibits RNA, DNA and protein synthesis	*Uragoga ipecacuanha*
104	1207069	01500877	KYNURAMINE	C9H12N2O	164.21		alkaloid		urine of various animals
105	538986698	00240422	2-ACETYLPYRROLE	C6H7NO	109.13	1072-83-9	alkaloid	hepatoprotectant, organoleptic	*Valeriana officinalis, Camellia thea, Paeonia moutan, Lycium chinense ,Streptomyces* A-5071
106	538977757	01503815	CEVADINE	C32H49NO9	591.75	62-59-9	alkaloid	antihypertensive	*Veratrum alba;* contains 20% veratridine
107	538977679	01503662	VERATRIDINE	C37H53NO12	703.83	71-62-5	alkaloid	antihypertensive	*Veraturm alba*
108	538977926	01500647	VINCAMINE	C21H26N2O3	354.45	1617-90-9	alkaloid	vasodilator	*Vinca minor*
109	538986026	01500611	VINBLASTINE SULFATE	C46H60N4O13S	909.07	143-67-9, 865-21-4 [vinblastine]	alkaloid	antineoplastic, spindle poison	*Vinca rosea*
110	538988039	01505004	VINDOLINE	C25H32N2O6	456.54	2182-14-1	alkaloid	CNS stimulant/depressant; antihyperglycaemic	*Vinca rosea, V pusilla*
111	538977628	00310023	HYPOXANTHINE	C5H4N4O	136.11	68-94-0	alkaloid		widely distributed in the plant and animal kingdom
112	538986186	02300330	PTERIN-6-CARBOXYLI C ACID	C7H5N5O3	207.15		alkaloid		widespread in animal kingdom; metabolite of pteroylglutamate
113	538977629	01503007	BETAINE HYDROCHLORIDE	C5H12ClNO2	153.61	590-46-5, 141-58-2 [replaced], 107-43-7 [betaine]	alkaloid	antiarteriosclerotic, hypolipaemic, hepatoprotectant	widespread in fungi and plants
114	538986432	00500071	LYCOPODINE PERCHLORATE	C16H26ClNO5	347.84		alkaloid		widespread in Lycopodium spp.
115	1207068	01500874	LEUCOPTERIN	C6H5N5O3	195.14		alkaloid		wing pigment of many butterflies, e.g., Pieris brassicae and Gonopteryxrhamni
116	1048	01500764	KINETIN	C10H9N5O	215.22	525-79-1	alkaloid	auxin, plant growth regulator, plant cell division promotor	yeast
117	538986097	01504176	RHETSININE	C19H17N3O2	319.37	526-43-2	alkaloid		*Zanthoxylum rhetsa, Evodia rutaecarpa*
118	538977605	00310001	ACONITIC ACID	C6H6O6	174.11	585-84-2	alkane		*Aconitum and Achillea* spp.
119	538988055	01505190	L(+/−)-ALLIIN	C6H11NO3S	177.22	556-27-4(−)	alkane	antibacterial,	*Allium* spp.

								antioxidant	
120	538987961	01505014	DIALLYL TRISULFIDE	C6H10S3	178.34	2050-87-5	alkane	antineoplastic	*Allium* spp.
121	538988028	01505174	GARLICIN	C6H10S2	146.27	2179-57-9	alkane	antineoplastic, antibacterial, apoptosis inducer, insecticide	*Allium* spp., *Descurainia sophia*
122	538986706	01505293	DIALLYL SULFIDE	C6H10S	114.21	592-88-1	alkane	anitbacterial, antifungal, antineoplastic, antihypercholesterol aemic, hepatoprotectant	*Allium* spp., *Wasabia japonica*
123	538986148	01504206	BOVINOCIDIN (3-nitropropionic acid)	C3H5NO4	119.08	504-88-1	alkane	antineoplastic	*Aspergillus, Streptomyces* spp.*& other microorganisms*
124	538985351	01501193	ERYSOLIN	C6H11NO2S2	193.29	504-84-7	alkane	antiproliferative	cabbage
125	538985941	00201227	NONIC ACID	C9H16O4	188.23		alkane		hydroysis product of actinonin
126	538986493	01504910	DIHYDROJASMONIC ACID, METHYL ESTER	C13H22O3	226.32	24851-98-7	alkane	plant growth regulator	Jasminium spp. and Vicia faba
127	538985923	01504104	DIHYDROJASMONIC ACID	C12H20O3	212.29	98674-52-3	alkane	plant growth regulator	*Jasminium* spp. *and Vicia faba*
128	538977946	01800177	PENICILLIC ACID	C8H10O4	170.17		alkane	antibacterial	*Penicillium* spp.
129	44	01500648	AZELAIC ACID	C9H16O4	188.23	123-99-9	alkane	antiacne, antiproliferative agent	*rancid fats and Lycopodium* spp.
130	538976370	01502236	ABRINE (L)	C12H14N2O2	218.26	526-31-8	amino acid	insecticide, antiinflammatory, antiopthalmic	*Abrus pecatorius*
131	538977386	01502183	DIPROTIN A	C17H31N3O4	341.45	90614-48-5	amino acid	dipeptidyl peptidase inhibitor, HIV inhibitor	*Bacillus cereus*
132	1205994	01500833	CANAVANINE	C5H12N4O3	176.18	543-38-4	amino acid	NO synthase inhibitor	*Canavalia ensiformis*
133	1210710	01500855	CITRULLINE	C6H13N3O3	175.19	627-77-0	amino acid		*Citrullis vulgaris and the alga Grateloupia filicina*
134	538978174	02300228	KAINIC ACID	C10H15NO4	213.24	487-79-6	amino acid	glutamate receptor agonist, anthelmintic	*Digenia simplex*
135	538977613	00310008	DJENKOLIC ACID	C7H14N2O4S2	254.33	498-59-9	amino acid		djenkol bean (*Pithecolobium lobatum*)
136	1207070	01500879	KYNURENINE	C10H12N2O3	208.22		amino acid		mamalian urine

137	538977498	01502262	NOPALINE	C11H20N4O6	304.31		amino acid		metabolite of plant tumors
138	1206022	01500869	MIMOSINE	C8H10N2O4	198.18		amino acid	depilatory agent	*Mimosa and Leucena* spp.
139	1205988	01500711	ALBIZZIINE	C4H9N3O3	147.13	585-23-9	amino acid	glutamase inhibitor	*Mimosaceae*
140	538986458	01400136	N-METHYLISOLEUCINE	C7H15NO2	145.20	5125-98-8	amino acid		*Phasealus vulgaris*
141	538977944	01502248	GLUTATHIONE	C10H17N3O6S	307.33	70-18-8	amino acid	antioxidant	plant and animal tissue
142	538986435	00501000	D,L-threo-3-HYDROXYASPARTIC ACID	C4H7NO5	149.10		amino acid	L-aspartate beta-carboxylase inhibitor	*Streptomyces* spp., *Arthrinium phaeospermum*, *Dactylosporangium aurantiacum*
143	538986628	01505254	THEANINE	C7H14N2O3	174.20	3081-61-6	amino acid		Thea sinensis
144	538986745	01505302	GENETICIN	C20H44N4O18S2	692.72	108321-42-2, 49863-47-0(base)	aminoglycoside	antibacterial	*Micromonospora* spp.
145	538985471	00200022	AKLAVINE HYDROCHLORIDE	C30H36ClNO10	606.08	60504-57-6 (aklavine)	anthraquinone	antibacterial, antineoplastic	*Actinomyces* spp.
146	538977544	00300545	CHRYSOPHANOL	C15H10O4	254.24	481-74-3	anthraquinone		*Cassia and Rumex* spp.
147	1210682	01500817	CARMINIC ACID	C22H20O14	508.40	1260-17-9	anthraquinone		Dactylopius coccus (cochineal)
148	538978228	01504060	EMODIC ACID	C15H8O7	300.23	578-45-5	anthraquinone	cathartic, purgative	*Penicillium cyclopium, Calopaca ferruginea*
149	538986510	00211473	1,4,5,8-TETRAHYDROXY-2,6-DIMETHYLANTHROQUINONE	C16H12O6	300.27	19079-10-8	anthraquinone		*Phoma terrestris and Curvularia lunata*
150	538986710	01505300	PURPURIN	C14H8O5	256.22	81-54-9	anthraquinone	xanthin oxidase inhibitor, irritant	*Rubia and Gallium* spp.
151	538977517	00210850	ALIZARIN	C14H8O4	240.22	72-48-0	anthraquinone	antimutagen	*Rubia tinctorum*
152	538986567	00201606	RUTILANTINONE	C22H20O9	428.40	21288-61-9	anthraquinone	coccidiostat	*Streptomyces species*
153	538986509	00201604	PYRROMYCIN	C30H35NO11	585.61	668-17-7	anthraquinone	antibacterial	*Streptomyces* spp.
154	538978137	01504070	PHYSCION	C16H12O5	284.27	521-61-9	anthraquinone	antibacterial, cathartic	*Xanthoria lichens, Rumex* spp. *and various Aspergillus* spp.
155	538985513	01504078	SENNOSIDE A	C42H38O20	862.76	81-27-6	anthraquinone	cathartic	*Cassia & Rheum* spp.
156	538986501	00210925	METHYL ORSELLINATE	C9H10O4	182.18	3187-58-4	aromatic		*lichens and lichen acids*
157	538988034	00200446	METHYLXANTHOXYLIN	C11H14O4	210.23	23121-32-6	aromatic		*Acradenia franklinii, Eugenia jambolana*
158	538986177	00210568	COUMARINIC ACID METHYL ETHER	C10H10O3	178.19		aromatic		*aglycone Melilotus alba, Dipteryx odorata*

159	538986457	01400131	HAEMATOMMIC ACID	C9H8O5	196.16	479-25-4	aromatic		*Alectoria* spp., *Haematomma* spp. *and Lethariella* spp.
160	538986695	00231070	p-HYDROXYCINNAM ALDEHYDE	C9H8O2	148.16	2538-87-6	aromatic		*Alpinia galanga, Sarcophyte sanguinea*
161	538977831	01503705	ANETHOLE	C10H12O	148.21	4180-23-8	aromatic	expectorant, gastric stimulant, insecticide	anise, fennel and other plant oils
162	538977595	00200110	ANTIAROL	C9H12O4	184.19	642-71-7	aromatic		*Antiaris toxicaria*
163	538986090	01504180	ASARININ (−)	C20H18O6	354.36	13079-95-3	aromatic	antibacterial (tuberculostatic)	*Asarum & Xanthoxylum* spp.
164	538977600	00210072	METHYLORSELLINIC ACID, ETHYL ESTER	C11H14O4	210.23		aromatic		Aspergillus silvaticus
165	538986015	00200640	3-METHYLORSELLINI C ACID	C9H10O4	182.18	4707-46-4	aromatic		Aspergillus terreus
166	538985933	00211126	DIPHENYLUREA	C13H12N2O	212.25	102-07-8	aromatic		coconut milk
167	538985272	01500296	EUGENOL	C10H12O2	164.21	97-53-0	aromatic	analgesic (topical), antiseptic, antifungal	common in plant essential oils
168	538976323	00300001	ORSELLINIC ACID	C8H8O4	168.15	480-64-8	aromatic		common lichen constituent
169	538986549	00290032	SINAPIC ACID METHYL ETHER	C12H14O5	238.24	90-50-6	aromatic		common plant constituent
170	538977663	01505345	CURCUMIN	C21H20O6	368.39	458-37-7	aromatic	antiedemic, antiinflammatory, bile stimulant; antibacterial, antifungal, lipo/cyclooxygenase inhibitor	*Curcuma* spp.
171	538986377	01400164	2-HYDROXY-3,4-DIME THOXYBENZOIC ACID	C9H10O5	198.18		aromatic	prostaglandin synthetase inhibitor	*Dalbergia odorifera*
172	538977799	00211458	3,4,5-TRIMETHOXYCIN NAMALDEHYDE	C12H14O4	222.24		aromatic		*Dalbergia spruceana*
173	538986420	00200208	ASARYLALDEHYDE	C10H12O4	196.20	4460-86-0	aromatic	fly attractant	*Daucus carota, Acorus calamus, Asarum europaeum*
174	538978232	00210599	2,5-DIHYDROXY-3,4-DI METHOXY-4′-ETHOXY BENZOPHENONE	C17H18O6	318.33		aromatic		derivative
175	538977911	00300157	EUGENYL BENZOATE	C17H16O3	268.32		aromatic		derivative
176	538985348	00300423	DIFUCOL	C18H22O6	334.37	14262-07-8	aromatic		derivative

			HEXAMETHYL ETHER						
177	538987953	01505006	DIHYDROMYRISTICIN	C11H14O3	194.23	607-91-0	aromatic	GSH transferase inducer	derivative; myristicin
178	538985978	00330085	4-NONYLPHENOL	C15H24O	220.36	25154-52-3	aromatic	weevil pheromone, shows estrogenic activity	elephant secretant
179	538986681	00212061	PYROCATECHUIC ACID	C7H6O4	154.12	303-38-8	aromatic	antioxidant	*Erythraea centaurium, Gentiana lutea*
180	538978056	00210752	EUDESMIC ACID	C10H12O5	212.20	118-41-2	aromatic		*eucalyptus oil*
181	538986375	00240740	4-ACETOXYPHENOL	C8H8O3	152.15	3233-32-7	aromatic	antioxidant	*Ferulago aucheri, Salvia yosgadensis*
182	3553	00201466	MANDELIC ACID, METHYL ESTER	C9H10O3	166.18		aromatic		*free acid found in Poria* spp.
183	538978109	01504019	GOSSYPOL	C30H30O8	518.57	303-45-7	aromatic	antispermatogenic, antineoplastic, antiHIV	*Gossypium* spp.
184	538986164	01600964	*N*-METHYLANTHRANILIC ACID	C8H9NO2	151.17	119-68-6	aromatic		grapefruit peel oil
185	538977564	00300533	ISOSAFROLE	C10H10O2	162.19	120-58-1	aromatic		*Illicium religiosum*
186	538978054	00210369	GALLIC ACID	C7H6O5	170.12	149-91-7	aromatic	antineoplastic, astringent, antibacterial	insect galls
187	1210756	01502206	NORDIHYDROGUARETIC ACID	C18H22O4	302.37		aromatic	lipoxygenase inhibitor, antioxidant	*Larrea divaricata*
188	538986507	00211009	ETHYL EVERNINATE	C11H14O4	210.23	6110-36-7	aromatic		lichen acids
189	538986505	00210924	METHYL EVERNINATE	C10H12O4	196.20	520-43-4	aromatic		lichens and lichen acids
190	538977966	01503992	CONIFERYL ALCOHOL	C9H10O3	166.18	458-35-5	aromatic		lignan product; common in plants as glycosides
191	538986696	00231074	o-VERATRALDEHYDE	C9H10O3	166.18	86-51-1	aromatic		*Machaerium kuhlmannii*
192	538986157	01600919	3-METHOXYCATECHOL	C7H8O3	140.14	934-00-9	aromatic		*Machaerium kuhlmanni*
193	1205857	00300146	VULPINIC ACID	C19H14O5	322.32	521-52-8	aromatic	antiinflammatory, antibacterial, plant growth inhibitor	numerous lichens, e.g. *Letharia vulpina*
194	538987939	01505117	ESTRAGOLE	C10H12O	148.21	140-67-0	aromatic	insect atttractant, skin irritant, carcinogen	numerous plant essential oils
195	538977558	00201727	EVERNINIC ACID	C8H8O5	184.15	570-10-5	aromatic		oak moss lichen
196	538985974	00210800	CHLOROGENIC ACID	C16H18O9	354.32	327-97-9	aromatic	antioxidant, free	occurence in many

								radical scavenger	plants
197	538986280	01601021	PAEONOL	C9H10O3	166.18	552-41-0	aromatic	antibacterial	*Paeonia montan, Xanthorrhoea* spp.
198	538986459	01400156	ISOPEONOL	C9H10O3	166.18	493-33-4	aromatic		*Paeonia* spp.
199	538986159	00211066	2-METHOXYRESORCI NOL	C7H8O3	140.14	29267-67-2	aromatic		*Peltophorum africanum*
200	4	00100616	ANGOLENSIN (R)	C16H16O4	272.30	4842-48-2	aromatic		*Pericopsis and Pterocarpus* spp.
201	538986498	00390001	APIOLE	C12H14O4	222.24	523-80-8	aromatic	antipyretic, diuretic, insecticide	*Petroselimum* spp., *Anethum graveolens*
202	538987985	01505130	3,4-DIMETHOXYCINN AMIC ACID	C11H12O4	208.22	2316-26-9	aromatic		*Piper methysticum, Veronica virginica*
203	538986175	00210567	DIMETHYLCAFFEIC ACID	C11H12O4	208.22	14737-89-4	aromatic		*Piper methysticum, Veronica virginica*
204	538986569	00200427	PISCIDIC ACID	C11H12O7	256.21	35388-57-9	aromatic		*Piscidia piscipula*
205	538985924	01504105	TANNIC ACID	C76H52O46	1701.23	1401-55-4	aromatic	nonspecific enzyme/receptor blocker	principal constituent of tree galls, esp Quercus spp.;
206	538986565	00203008	JUAREZIC ACID	C11H10O2	174.20	1552-94-9	aromatic		*propolis and Populus* spp.
207	538986777	01505349	4-*O*-METHYLPHLORA CETOPHENONE	C9H10O4	182.18	7507-89-3	aromatic	antifungal	*Prunus domesticus*
208	538986776	00300604	PHLORACETOPHENO NE	C8H8O4	168.15	480-66-0	aromatic		*Prunus domesticus*
209	717	00200002	ORSELLINIC ACID, ETHYL ESTER	C10H12O4	196.20	2524-37-0	aromatic		Roccella and Lecanora lichens
210	538977503	00205077	ISOFERULIC ACID	C10H10O4	194.19		aromatic		roots of *Cimicifuga racemosa*
211	1210900	01502094	ROSMARINIC ACID	C18H16O8	360.32	537-15-5	aromatic	antiinflammatory, antithrombotic, antiplatelet, cytostatic, antiviral	*Rosmarinus officinalis, Melissa officinalis, Momordica balsamina, Mentha piperita, Salvia officinalis, Teucrium scorodonia, Sanicula europaea, Coleus blumei, Thymus* spp.
212	538977524	01503620	SAFROLE	C10H10O2	162.19	94-59-7	aromatic	anesthetic (topical) and antiseptic, pediculicide	*sassafras officinale*
213	538977888	00100577	SAFROLGLYCOL	C10H12O4	196.20		aromatic		sassafras root
214	538986502	00206050	ORSELLINIC ACID DIMETHYL ETHER	C10H12O4	196.20	3686-57-5	aromatic		semisynthetic

215	538977591	00200759	IRETOL	C7H8O4	156.14		aromatic		semisynthetic
216	538986083	00300610	ACETOSYRINGONE	C10H12O4	196.20	2478-38-8	aromatic	insect attractant, plant hormone	several commercial woods
217	538988101	00212097	ONONETIN	C15H14O4	258.28	487-49-0	aromatic		*Trifolium subterraneum*
218	538986022	01503006	BENZYL ISOTHIOCYANATE	C8H7NS	149.22	622-78-6	aromatic	antineoplastic, antibacterial, antifungal	*Tropaeolum majus, Lepidium sativum and other Cruciferae*
219	1206968	00205071	HAEMATOMMIC ACID, ETHYL ESTER	C11H12O5	224.22	39503-14-5	aromatic		various lichens, *e.g., Evernia* spp., *Parmelia* spp.
220	538985959	00212140	VERATRIC ACID	C9H10O4	182.18	93-07-2	aromatic		*Verbascum thapsus, Sabadilla officinalis, Stephania abyssinica*
221	538980168	00211307	GENTISIC ACID	C7H6O4	154.12	490-79-9	aromatic	analgesic, antiinflammatory	widely distributed in higher plants and *Penicillium* spp.
222	538976400	01502245	ELLAGIC ACID	C14H6O8	302.20	476-66-4	aromatic	hemostatic, antineoplastic, antimutagenic	widely distributed in higher plants
223	18	01501017	FERULIC ACID	C10H10O4	194.19	1135-24-6	aromatic	antineoplastic, choleretic, food preservative	widely distributed in plants
224	538977968	01503987	CAFFEIC ACID	C9H8O4	180.16	331-39-5	aromatic		widespread in plants
225	538986158	00200441	XANTHOXYLIN	C10H12O4	196.20	90-24-4	aromatic		*Xanthoxylium* spp., *Artemisia brevifolia*
226	20	00300547	PHLORIDZIN	C21H24O10	436.42	60-81-1	aromatic glycoside	induces experimental glucosuria, antifeedant	*Rosaceae* spp.
227	538977910	00300062	METAMECONINE	C10H10O4	194.19		benzofuran		*Accacia crombei*
228	538977646	00310041	VISNAGIN	C13H10O4	230.22	82-57-5	benzofuran		*Ammi visnaga*
229	1201361	00300007	EUPARIN	C13H12O3	216.24	532-48-9	benzofuran		*Eupatorium macculatum*
230	538977947	00300147	USNIC ACID	C18H16O7	344.32	125-46-2	benzofuran	antibacterial	*Usnea, Cladonia and other lichen* spp.
231	538977961	00290030	CEAROIN	C14H12O4	244.25	52811-37-7	benzophenone		*Dalbergia* spp.
232	538986779	00200425	2,3-DIHYDROXY-4-METHOXY-4′-ETHOXYBENZOPHENONE	C16H16O5	288.30		benzophenone		derivative
233	1210684	01500819	BERGENIN	C14H16O9	328.28	477-90-7	benzopyran		Bergenia spp.
234	2571	00210201	DIHYDROMUNDULONE	C26H30O6	438.53		benzopyran		borohydride reduction of mundulone
235	538978204	01504036	3-DESHYDROXYSAPP	C19H22O5	330.38		benzopyran		derivative

			ANOL TRIMETHYL ETHER						
236	1205854	00210203	DIHYDROMUNDULETONE	C25H28O6	424.50		benzopyran		derivative
237	538986240	01600365	SAPPANONE A TRIMETHYL ETHER	C19H18O5	326.35		benzopyran		derivative *Caesalpinia sappan*
238	538986241	01600480	TETRAHYDROSAPPANONE A TRIMETHYL ETHER	C19H22O5	330.38		benzopyran		derivative *Caesalpinia sappan*
239	538986239	01600587	TUBAIC ACID	C12H12O4	220.23	25277-45-6	benzopyran		*Derris eliptica*; also degradation product of rotenone.
240	1205437	00300061	EUPATORIOCHROMENE	C13H14O3	218.25	19013-03-7	benzopyran	antifungal	*Eupatorium rugosum*
241	1205152	00200012	BRAZILIN	C16H14O5	286.29	474-07-7	benzopyran		*Haematoxylin campechianum*
242	1205483	00200010	HAEMATOXYLIN	C16H14O6	302.29	517-28-2	benzopyran		*Haemotoxylon campechianum*
243	21284	01504165	OSTHOL	C15H16O3	244.29	484-12-8	benzopyran		*Imperatoria ostruthium*& other Umbelliferae
244	538976409	01502253	HEMATEIN	C16H12O6	300.27	475-25-2	benzopyran		logwood, *Haematoxylon* spp.
245	538977751	00201654	MAACKIAIN	C16H12O5	284.27		benzopyran		*Maackia* spp.
246	1205498	00100743	HOMOPTEROCARPIN	C17H16O4	284.31	606-91-7	benzopyran		mp 82-84 C; *Pterocarpus santalinus*
247	538977858	00201156	MEDICARPIN	C16H14O4	270.29		benzopyran		widespread in Fabaceae
248	538988014	01505160	HINOKITIOL	C10H12O2	164.21	499-44-5	benztropolone	antifungal, insecticide, plant growth inhibitor, mettaloprotease inhibitor, DNA synthesis inhibitor, apoptosis inducer	*Chamaecyparis obtusa*, *Juniperus* spp., *Thuja* spp.
249	2857	00210505	PURPUROGALLIN	C11H8O5	220.18	569-77-7	benztropolone	xanthine oxidase inhibitor, antioxidant	gall of Dryophanta divisa
250	538977854	00200111	THEAFLAVIN	C29H24O12	564.51	4670-05-7	benztropolone	antioxidant	pigment in black tea
251	1210788	01500836	CEPHALOSPORIN C SODIUM	C16H20N3NaO8S	437.41	61-24-5	betalactam	antibacterial	*Cephalosprium acremonium*
252	538986464	01500352	INOSITOL	C6H12O6	180.16	87-89-8	carbohydrate	growth factor	lipotropic polyol

									widely distributed in plants and animals
253	538977606	00310002	ADONITOL	C5H12O5	152.15	488-81-3	carbohydrate		*Adonis* spp.
254	538977513	00300539	ARBUTIN	C12H16O7	272.26	497-76-7	carbohydrate		*Berginia crassifolia; also in Pyrus and Vaccinium* spp.
255	8	01501211	MUCIC ACID	C6H10O8	210.14	526-99-8	carbohydrate		brown algae, various fruits and fungi
256	538977623	00310018	QUINIC ACID	C7H12O6	192.17	77-95-2	carbohydrate		*Cinchona* spp.
257	538976419	01502256	SHIKIMIC ACID	C7H10O5	174.15	138-59-0	carbohydrate		common constituent in plants
258	538977500	01502261	QUEBRACHITOL	C7H14O6	194.19	642-38-6	carbohydrate		common in dicotyledons
259	538977953	01503989	GLUCITOL-4-GUCOPY ANOSIDE	C12H24O11	344.32		carbohydrate		derivative cotton
260	1207054	00240437	PERSITOL HEPTAACETATE	C21H30O14	506.46	19147-10-5	carbohydrate		derivative of perseitol
261	538976375	01502231	CELLOBIOSE (D[+])	C12H22O11	342.30	528-50-7	carbohydrate		enzymatic hydrolysis of cellulose
262	538977567	00300531	MELEZITOSE	C18H32O16	504.45		carbohydrate		honey & plant exudates
263	538986173	00211539	ARABITOL(D)	C5H12O5	152.15	488-82-4	carbohydrate		Lecanora sordida & other lichen & fungi
264	538986019	01504124	LINAMARIN	C10H17NO6	247.25	554-35-8	carbohydrate		*Linum usitatissimum, Manihot utilissimus*
265	538985658	03100024	INOSINE	C10H12N4O5	268.23	58-63-9	carbohydrate	cell function activator, cardiotonic	meat extracts, sugar beet, *Bacillus subtilis, E. coli, Saccharomyces cerevisiae, Fusarium* spp.
266	538977510	00202130	PERSEITOL	C7H16O7	212.20	527-06-0	carbohydrate		*Persia* spp.
267	538976396	01502244	AMYGDALIN	C20H27NO11	457.44	29883-15-6	carbohydrate	antiinflammatory, experimental antineoplastic	*Rosaceae* spp.
268	538976417	01502255	SALICIN	C13H18O7	286.28	138-52-3	carbohydrate	analgesic, antipyretic	*Salix* spp.
269	538978131	01504066	LOGANIN	C17H26O10	390.39	18524-94-2	carbohydrate		*Strychnos nux-vomica, Menyanthes trifoliata*
270	538978132	01504071	LOGANIC ACID	C16H24O10	376.36	22255-40-9	carbohydrate		*Swertia caroliniensis, Dipsacus asperoides*
271	538986226	00200258	2′,4′-DIHYDROXYCHA LCONE 4′-GLUCOSIDE	C21H22O8	402.40		chalcone	anthelmintic & antiulcerocenic	aglycone Flamingia chappar, Acacia

									neovernicosa
272	538988007	01505152	2′,4′-DIHYDROXY-4-METHOXYCHALCONE	C16H14O4	270.29	81674-91-1	chalcone		*Bauhinia manca*
273	538986390	00211475	4′-METHOXYCHALCONE	C16H14O2	238.29	22966-19-4	chalcone		*Citrus limon*
274	538988003	01505148	2′,4′-DIHYDROXY-3,4-DIMETHOXYCHALCONE	C17H16O5	300.31		chalcone		*Iryanthera polyneura*
275	538988008	01505153	2′,3-DIHYDROXY-4,4′,6′-TRIMETHOXYCHALCONE	C18H18O6	330.34	38186-71-9	chalcone		*Merrillia caloxylon*
276	538986418	00200123	2′,beta-DIHYDROXYCHALCONE	C15H12O3	240.26		chalcone		*Primula pulverulenta.*
277	21	00300554	PHLORETIN	C15H14O5	274.28	60-82-2	chalcone		*Prunus* spp.
278	538986391	00200407	4′-HYDROXYCHALCONE	C15H12O2	224.26	2657-25-2	chalcone		Shorea robusta aglycone
279	538987995	01505140	2′,4-DIHYDROXY-3,4′,6′-TRIMETHOXYCHALCONE	C18H18O6	330.34	112572-59-5	chalcone		*Viscum album* (glucoside)
280	538986112	01504200	ISOLIQUIRITIGENIN	C15H12O4	256.26	961-29-5	chalcone	aldose reductase inhibitor, antineoplastic, antiinflammatory	widespread in Fabaceae
281	2465	00210866	KHELLIN	C14H12O5	260.25	82-02-0	chromone	vasodilator	*Amni visnaga Lam., Umbelliferae*
282	538986223	00200484	DEOXYSAPPANONE B 7,4′-DIMETHYL ETHER	C18H18O5	314.34		chromone		*Caesalpinia sappan*
283	538986210	00201331	DEOXYSAPPANONE B TRIMETHYL ETHER	C19H20O5	328.37		chromone		*Caesalpinia sappan*
284	538986209	00201136	SAPPANONE A DIMETHYL ETHER	C18H16O5	312.33		chromone		*Caesalpinia sappan*
285	538986352	00210658	DEHYDROVARIABILIN	C17H14O4	282.30		chromone		*Dalbergia variabilis*
286	538978147	00200457	ANHYDROBRAZILIC ACID	C12H10O5	234.21		chromone		derivative
287	538986224	00201342	DEOXYSAPPANONE B 7,3′-DIMETHYL ETHER ACETATE	C20H20O6	356.38		chromone		derivative Caesalpinia sappan
288	1211882	00200447	EUGENITOL	C11H10O4	206.20	491-48-5	chromone		*Eugenia caryophyllata*
289	636	00200449	ISOEUGENITOL	C11H10O4	206.20	479-06-1	chromone		*Eugenia caryophyllata*
290	1201554	00100528	PEUCENIN	C15H16O4	260.29	578-72-3	chromone		*Peucedanum ostruthium, Ptaeroxylon*

									obliquum
291	1201627	00100513	PTAEROXYLIN	C15H14O4	258.28	14729-11-4	chromone		*Ptaeroxylon obliquum, Cedrelopsis grevei*
292	1201322	00100529	HETEROPEUCENIN, METHYL ETHER	C16H18O4	274.32	26213-95-6	chromone		*Ptaeroxylon obliquum, Harrisonia perforate ,Neochamaelea pulverulenta.*
293	538987986	01505131	ESCULIN MONOHYDRATE	C15H18O10	358.30	531-75-9	coumarin	antiinflammatory	*Fraxinus* spp.
294	21276	01504162	MARMESIN	C14H14O4	246.27	13849-08-6	coumarin		*Aegle marmelos, Amni majus*
295	1210715	01500899	ESCULETIN	C9H6O4	178.15	305-01-1	coumarin	antifungal	*Aesculus and Solanaceae* spp.
296	538986174	00211538	3-HYDROXYCOUMARI N	C9H6O3	162.15	939-19-5	coumarin		*Alyxia lucida*
297	21273	01504161	DIHYDROSAMIDIN	C21H24O7	388.42		coumarin		*Amni visnaga*
298	21281	01504163	MARMESIN ACETATE	C16H16O5	288.30		coumarin		*Amyris elemifera*
299	538977929	01500729	4-METHYLESCULETIN	C10H8O4	192.17		coumarin		*analog of esculetin*
300	2068	00102076	IMPERATORIN	C16H14O4	270.29	482-44-0	coumarin	anticonvulsant, antiinflammatory	*Angelica &*other Umbelliferaceae.
301	538986697	00231084	UMBELLIFERONE	C9H6O3	162.15	93-35-6	coumarin	antifungal, phytoalexin	*Angelica, Artemisia, Coronilla, Ferula and Ruta* spp.
302	538977549	01500707	CITROPTEN	C11H10O4	206.20	487-06-9	coumarin	photosensitizing agent	Bergamot oil
303	1201717	00100609	XANTHYLETIN	C14H12O3	228.25	553-19-5	coumarin		*Brosimum rubescens, Ruta, Boenninghausenia, Flindersia, Zanthoxylum, and Luvunga* spp.
304	538988030	01505176	AURAPTENE	C19H22O3	298.39	495-02-3	coumarin	antineoplastic, apoptosis inducer	*Citrus aurantium, Feronia elephantum, Aegle marmelos, Libanotis intermedia*
305	538987992	01505137	BERGAPTOL	C11H6O4	202.17	486-60-2	coumarin		Citrus spp.
306	538986460	01400208	COUMARIN	C9H6O2	146.15	91-64-5	coumarin	antineoplastic, antiinflammatory, antihyperglycaemic	*Coumarouna odorata,* tonka beans, lavender oil
307	538977553	00300540	HYMECROMONE METHYL ETHER	C11H10O3	190.20	2555-28-4	coumarin		*Dalbergia volubilis, Eupatorium pauciflorum*
308	538977890	00100572	FRAXIDIN METHYL ETHER	C12H12O5	236.23		coumarin		derivative

309	538985937	00240673	ROBUSTIC ACID	C22H20O6	380.40	5307-59-5	coumarin		*Derris robusta*
310	538977581	00211566	SCANDENIN DIACETATE	C30H30O8	518.57		coumarin		*Derris scandens*
311	538978162	00300348	SCANDENIN	C26H26O6	434.49	5084-00-4	coumarin		*Derris scandens*
312	21278	01504164	FELAMIDIN	C21H18O5	350.37	17559-85-2	coumarin		*Ferulago meoides*
313	1201446	00300032	ISOBERGAPTENE	C12H8O4	216.20	482-48-4	coumarin		Heracleum and other Umbelliferaceae; mp 223–224 C
314	1201681	00300005	SPHONDIN	C12H8O4	216.20	483-66-9	coumarin		Heracleum maximum root; mp 191–192 C
315	1201684	00300013	PIMPINELLIN	C13H10O5	246.22	131-12-4	coumarin	GABA receptor antagonist, phototoxin	Heracleum maximum root; mp 118–119 C
316	1201447	00300012	ISOPIMPINELLIN	C13H10O5	246.22	482-27-9	coumarin		Hercaleum maximum; mp 150–153 C dimorphic (142-145 C)
317	538978163	00210874	HERNIARIN	C10H8O3	176.17	551-59-9	coumarin		*Herniaria* spp.
318	21289	01504158	LOMATIN	C14H14O4	246.27		coumarin		Lomatium nuttallii, Nardostachys jatamansi
319	26	00300228	LONCHOCARPIC ACID	C26H26O6	434.49	5490-47-1	coumarin		*Lonchocarpus and Derris* spp.
320	538977976	00240862	KUHLMANNIN	C17H14O5	298.30		coumarin		*Manchaerium* spp.
321	3617	00201602	PACHYRRHIZIN	C19H12O6	336.30	10091-01-7	coumarin	insecticide	Pachyrrhizus erosus; mp 206–207 C
322	21277	01504168	PEUCEDANIN	C15H14O4	258.28	133-26-6	coumarin		Peucedanum spp.
323	538976346	00100101	PRENYLETIN	C14H14O4	246.27	15870-91-4	coumarin		*Ptaeroxylon obliquum*
324	21274	01504160	PTERYXIN	C21H22O7	386.41	13161-75-6	coumarin	muscle relaxant	*Pteryxia* spp., *Amni ursina*
325	538976418	01502242	SCOPOLETIN	C10H8O4	192.17	92-61-5	coumarin	NO synthesis (inducible) inhibitor, anticoagulant	*Scopolia* spp.
326	21275	01504159	SELINIDIN	C19H20O5	328.37	19427-82-8	coumarin		*Selinium vaginatum*
327	1201561	00100540	OBLIQUIN	C14H12O4	244.25		coumarin		*sneezewood or umtati, Ptaeroxylon Obliquum*
328	538985623	00300546	BERGAPTEN	C12H8O4	216.20	484-20-8	coumarin	antipsoriatic, antiinflammatory	widespread in Umbelliferae
329	1210717	01500901	AESCULIN	C15H16O9	340.29	531-75-9	coumarin glycoside	antiinflammatory	*Aesculus hippocastanum, Fraxinus* spp.
330	538986511	00240942	ARTHONIOIC ACID	C29H36O9	528.60	25556-24-5	depside		*Arthonia impolita*
331	538986581	01505218	BAEOMYCESIC ACID	C19H18O8	374.35	644-66-6	depside		*Baeomyces* spp.

332	1201518	00200070	LECANORIC ACID	C16H14O7	318.29	480-56-8	depside		Common constituent of lichens
333	1201033	00200034	ATRANORIN	C19H18O8	374.35	479-20-9	depside		Common lichen metabolite
334	538977554	00201343	EVERNIC ACID	C17H16O7	332.31	537-09-7	depside	plant growth regulator	*Evernia* spp., *Parmelia* spp. and other lichens
335	538985970	01504118	DIFFRACTAIC ACID	C20H22O7	374.39	436-32-8	depside		*Usnea* spp.
336	1201336	00200054	FUMARPROTOCETRA RIC ACID	C22H16O12	472.37	489-50-9	depsidone		Cetraria islandica
337	538976305	00300222	GANGLEOIDIN ACETATE	C20H16Cl2O8	455.25		depsidone		derivative of gangleodin (00200045)
338	538985955	00200035	GANGALEOIDIN	C18H14Cl2O7	413.21	55365-63-4	depsidone		Lecanora gangaleoides
339	1210792	00241150	LEOIDIN DIMETHYL ETHER	C20H18Cl2O7	441.27		depsidone		lichen constitutent
340	538976308	00200033	LEOIDIN	C18H14Cl2O7	413.21	105350-54-7	depsidone		lichen metabolite
341	1201517	00300018	LOBARIC ACID	C25H28O8	456.50		depsidone		lichens of the genus Stereocaulon and others
342	1207092	00201716	NORSTICTIC ACID	C18H12O9	372.29	571-67-5	depsidone	antibacterial	*Lobaria pulmonaria, Usnea japonica, Lecanora radiosa, Parmelia & Ramalina* spp.
343	1201682	00300006	STICTIC ACID	C19H14O9	386.32	549-06-4	depsidone		numerous lichens, e.g. *Parmelia sp.*
344	1205030	00300050	ABIENOL	C20H34O	290.49	17990-16-8	diterpene		*Abies* spp.
345	538977565	00300532	ANDROGRAPHOLIDE	C20H30O5	350.46	5508-58-7	diterpene		*Adrographis peniculata*
346	538977969	01503986	CAFESTOL ACETATE	C22H30O4	358.48	81760-48-7	diterpene		coffee bean oil
347	538980333	01503804	COLFORSIN	C22H34O7	410.51	66575-29-9	diterpene	adenylate cyclase activator, antiglaucoma, hypotensive, vasodilator	Coleus forskohlii; HL-362; L-75-1362B
348	538976369	01502229	ABIETIC ACID	C20H30O2	302.46	514-10-3	diterpene		common diterpene acid in conifers
349	370	00300058	EPI(13)TORULOSOL	C20H34O2	306.49	3650-30-4	diterpene		*Cryptomeria japonica and Larix sibirica*
350	538977909	00300060	8-HYDROXY-15,16-BIS NOR-11-LABDEN-13-O NE	C18H30O2	278.44		diterpene		derivative
351	538986270	01504234	18-AMINOABIETA-8,11 ,13-TRIENE SULFATE	C20H33NO4S	383.55		diterpene		derivative of abietic acid
352	538987901	00307033	3-HYDROXY-4-(SUCCI	C19H28O4	320.43		diterpene		derivative of

			N-2-YL)-CARYOLANE delta-LACTONE						caryophyllene
353	538987957	01505010	EUPHORBIASTEROID	C32H40O8	552.67	28649-59-4	diterpene		*Euphorbia lathyris, Macaranga tanarius*
354	538976405	00300021	GIBBERELLIC ACID	C19H22O6	346.38	77-06-5	diterpene		*Gibberella fujikuroi*
355	538988031	01505177	RUBESCENSIN A	C20H28O6	364.44	28957-04-2	diterpene	antibacterial, antineoplastic, insect growth inhibitor	*Isodon trichocarpus, I japonicus, Rabdosia* spp.
356	538986586	01502023	7-OXOCALLITRISIC ACID, METHYL ESTER	C21H28O3	328.46	155473-19-1 (acid)	diterpene		*Juniperus chinensis*
357	538978114	01504024	LAGOCHILIN	C20H36O5	356.51	23554-81-6	diterpene		*Lagochilus inebrians*
358	538976420	00300057	LARIXOL ACETATE	C22H36O3	348.53		diterpene		*Larix europaea*
359	538976421	00300056	LARIXOL	C20H34O2	306.49		diterpene		*Larix sibiricia*
360	3648	00201662	GRAYANOTOXIN I	C22H36O7	412.53	4720-09-6	diterpene		*Leucothoe grayana*
361	538987956	01505009	GERANYLGERANIOL	C20H34O	290.49	24034-73-9	diterpene	apoptosis inducer	linseed oil; widespread in plants
362	538977584	00100271	TOTARALOLAL	C20H28O2	300.44		diterpene		*Podocarpus* spp.
363	538977560	00100280	19-HYDROXYTOTARO L	C20H30O2	302.46	2288-33-7	diterpene		*Podocarpus* spp.
364	538977562	00100286	PODOTOTARIN	C40H58O2	570.91		diterpene		*Podocarpus* spp.
365	538977561	00100612	TOTAROL-19-CARBOX YLIC ACID, METHYL ESTER	C21H30O3	330.47		diterpene		*Podocarpus* spp.
366	538978083	00100287	TOTAROL	C20H30O	286.46	511-15-9	diterpene		*Podocarpus* spp., *Dacrydium cupressinum, Tetraclinis articulata and Thujopsis dolabrata*
367	905	00100267	TOTAROL ACETATE	C22H32O2	328.50		diterpene		*Podocarpus totara*
368	538988041	01505081	SALVINORIN B	C21H26O7	390.44	92545-30-7	diterpene	principal metabolite of salvinorin A	*Salvia divinorum*
369	538988040	01505080	SALVINORIN A	C23H28O8	432.47	83729-01-5	diterpene	k-opiod receptor agonist, psychotropic	Salvia divinorum
370	538986020	01504120	CARNOSIC ACID	C20H28O4	332.44	3650-09-7	diterpene		*Salvia* spp., *Rosmarinus officinalis*
371	853	00300003	SOLIDAGENONE	C20H28O3	316.44	23534-56-7	diterpene		Solidago canadensis L.; mp 133–134 C
372	538988047	01505034	BACCATIN III	C31H38O11	586.64	27548-93-2	diterpene		Taxus baccata
373	538980335	01503908	PACLITAXEL	C47H51NO14	853.93	33069-62-4	diterpene	antineoplastic	Taxus breviofolia
374	538977975	01504005	TRIPTOPHENOLIDE	C20H24O3	312.41	74285-86-2	diterpene		Tripterygium wilfordii
375	538988000	01505145	ERIODYCTOL	C15H12O6	288.26	552-58-9	flavan	expectorant	common flavan in

									numerous plant families
376	538985962	00240567	8-IODOCATECHIN TETRAMETHYL ETHER	C19H21IO6	472.28		flavan		derivative
377	2056	00210220	CATECHIN TETRAMETHYLETHER	C19H22O6	346.38		flavan		derivative
378	538977781	01600537	CATECHIN PENTAACETATE	C25H24O11	500.46		flavan		derivative
379	538986361	01504256	EPICATECHIN PENTAACETATE	C25H24O11	500.46		flavan		derivative
380	538977826	00210211	EPIAFZELECHIN TRIMETHYL ETHER	C18H20O5	316.36		flavan		derivative
381	2342	00205113	EPIGALLOCATECHIN	C15H14O7	306.27	970-74-1	flavan		green tea
382	538977685	01600561	LIQUIRITIGENIN DIMETHYL ETHER	C17H16O4	284.31	95753-46-1	flavan		*Holocarpha obconica*
383	538986677	00202178	EPIAFZELECHIN (2*R*,3*R*)(−)	C15H14O5	274.28	24808-04-6	flavan		*Larix sibirica, Actinidia chinensis, Juniperus communis, Cassia javanica*
384	538986065	01504154	PINOCEMBRIN	C15H12O4	256.26	36052-37-6	flavan	antiinflammatory	*Pinus, Prunus, Eucalyptus* spp.
385	538985689	01504084	DIHYDROROBINETIN	C15H12O7	304.26		flavan		*Robinia pseudoacacia*
386	538986630	01505256	SILIBININ	C25H22O10	482.45	22888-70-6	flavan	hepatoprotective agent, antioxidant	*Silybum marianum*
387	538977823	00210205	CIANIDANOL	C15H14O6	290.28	154-23-4	flavan	hepatoprotectant	tea and cocoa constituent; (+) catechin
388	2338	00210206	EPICATECHIN	C15H14O6	290.28	490-46-0	flavan	antioxidant	tea and cocoa constituent
389	538977936	01500746	NARINGENIN	C15H12O5	272.26	480-41-1	flavan	antiulcer, gibberellin antagonist	widely distributed in plants
390	80	01500765	NARINGIN	C27H32O14	580.55	10236-47-2	flavan glycoside	antihaemorrhagic, antiinflammatory	*Citrus* spp.
391	538978173	00211155	2,3-METHANO-7,2′-DI METHOXYFLAVANON E	C18H16O4	296.33		flavanone		derivative
392	538988086	01505382	5-HYDROXY-2′,4′,7,8-TE TRAMETHOXYFLAVO NE	C19H18O7	358.35	123316-61-0	flavone		*Citrus* spp., *Limnophilia rugosa*
393	101	01500721	7,4′-DIHYDROXYFLAV ONE	C15H10O4	254.24	2196-14-7	flavone	antioxidant	*Pterocarpus marsupium*

394	1035	01500719	7,2′-DIHYDROXYFLAVONE	C15H10O4	254.24	77298-66-9	flavone	antihaemorrhagic	*Primula* spp.
395	538988084	01505380	3,4′,5,6,7-PENTAMETHOXYFLAVONE	C20H20O7	372.38	4472-73-5	flavone		*Citrus* spp.
396	538988087	01505383	HEXAMETHYLQUERCETAGETIN	C21H22O8	402.40	1251-84-9	flavone		*Citrus* spp.
397	1033	01500717	6,4′-DIHYDROXYFLAVONE	C15H10O4	254.24	63046-09-3	flavone	antihaemorrhagic	*Cassia* spp. as glycoside
398	538977617	00310012	HESPERETIN	C16H14O6	302.29	520-33-2	flavone		aglycone of hesperidin (00310011)
399	538988002	01505147	3,5-DIHYDROXYFLAVONE	C15H10O4	254.24	6665-69-6	flavone		*Anthemis tinctoria* (glucoside)
400	538986014	01501012	3-HYDROXYFLAVONE	C15H10O3	238.25	577-85-5	flavone		cabbage
401	538977499	01502259	MORIN	C15H10O7	302.24	480-16-0	flavone	P450 and ATPase inhibitor	*Chlorophora tinctoria*
402	538977937	01500742	7,2′-DIMETHOXYFLAVONE	C17H14O4	282.30		flavone		*Citrus* spp.
403	538986649	01505268	NOBILETIN	C21H22O8	402.40	478-01-3	flavone		*Citrus* spp.
404	538988096	01505381	SINENSETIN	C20H20O7	372.38	2306-27-6	flavone		*Citrus* spp.
405	538986650	01505269	TANGERITIN	C20H20O7	372.38	481-53-8	flavone		*Citrus* spp., *Fortunella japonica*
406	538988044	01505030	DEMETHYLNOBILETIN	C20H20O8	388.38	2174-59-6	flavone		*Citrus, Sideritis, Heteropappus and Thymus* spp.; *Mentha piperita, Amaracus pampanini*
407	538986455	01600075	QUERCETIN PENTAMETHYL ETHER	C20H20O7	372.38		flavone		derivative
408	538986006	01500741	6,4′-DIMETHOXYFLAVONE	C17H14O4	282.30	54401-47-7	flavone		derivative Cassia spectabilis
409	538977603	00200833	ACACETIN DIACETATE	C20H16O7	368.35	5892-39-7	flavone		derivative of acacetin
410	538986003	01500731	EUPATORIN	C18H16O7	344.32	855-96-9	flavone	emetic	*Eupatorium* spp. and other Compositae
411	538988045	01505031	GARDENIN B	C19H18O7	358.35	2798-20-1	flavone		*Gardenia lucida; Brickellia, Citrus and Mentha* spp.
412	538986295	00200433	ISOGINKGETIN	C32H22O10	566.53	548-19-6	flavone	bradykinin antagonist	Ginkgo biloba leaves
413	538986052	00200436	GINKGETIN, K salt	C32H21KO10	604.62	481-46-9 (ginkgetin)	flavone		Ginkgo biloba leaves

414	538985971	01504115	HIERACIN	C15H10O7	302.24	1621-84-7	flavone		Ginkgo biloba, *Hieracium pilosella and Isoetes* spp.
415	3052	00201315	7,8-DIHYDROXYFLAV ONE	C15H10O4	254.24	38183-03-8	flavone	vascular protectant, antihaemorrhagic	*Godmania aesculifolia*
416	538988004	01505149	GERALDOL	C16H12O6	300.27	21511-25-1	flavone		*Lotus corniculatus, Tetragonolobus siliquosus, Anthyllis vulneraria, Trifolium subterraneum*
417	538987993	01505138	3,4′-DIHYDROXYFLAV ONE	C15H10O4	254.24	14919-49-4	flavone		*Millettia zechiana (Me ether)*
418	3603	00201581	SERICETIN	C25H24O5	404.47	42438-75-5	flavone		*Mundulea sericea, M suberosa*
419	538977609	00200846	APIGENIN	C15H10O5	270.24	520-36-5	flavone	antispasmodic, antineoplastic, topoisomerase I inhibitor	parsley seed
420	538987991	01505136	3′,6-DIHYDROXYFLAV ONE	C15H10O4	254.24	71592-46-6	flavone		*Pimelea decora* (di-Me ether)
421	538986013	01504132	6,3′-DIMETHOXYFLAV ONE	C17H14O4	282.30	79786-40-6	flavone		*Pimelia decora*
422	538977935	01500709	CHRYSIN	C15H10O4	254.24	480-40-0	flavone	diuretic	*Pinus, Scutellaria, and Ulnus* spp.
423	538986388	00201610	FLAVOKAWAIN B	C17H16O4	284.31	1775-97-9	flavone		*Piper, Alpinia & Myrica* spp.
424	538986011	01504130	3,7-DIHYDROXYFLAV ONE	C15H10O4	254.24	492-00-2	flavone		*Platymiscium praecox*
425	538977933	01500737	3,7-DIMETHOXYFLAV ONE	C17H14O4	282.30	20950-52-1	flavone		*Pongamia pinnata*
426	538987994	01505139	3′,4′-DIHYDROXYFLAV ONE	C15H10O4	254.24	4143-64-0	flavone		*Primula officinalis* (glucoside)
427	538986286	01500735	3′,4′-DIMETHOXYFLAV ONE	C17H14O4	282.30	4143-62-8	flavone		*Primula officinalis*
428	538986288	01500740	8,2′-DIMETHOXYFLAV ONE	C17H14O4	282.30		flavone		*Primula pulverulenta*
429	538987934	01501197	PRIMULETIN	C15H10O3	238.25	491-78-1	flavone		*Primula* spp.
430	538977934	01500738	5,2′-DIMETHOXYFLAV ONE	C17H14O4	282.30		flavone		*Primula* spp.
431	538977636	00310031	RHAMNETIN	C16H12O7	316.27	90-19-7	flavone		*Rhamnus cathartica*
432	538976403	01502247	FISETIN	C15H10O6	286.24	528-48-3	flavone	antioxidant	*Rhus and Acacia* spp.
433	538976387	00200499	ACACETIN	C16H12O5	284.27	480-44-4	flavone	antiinflammatory,	*Robinia pseudoacacia*

								spasmolytic agent, antioxidant	
434	538986367	00240958	4′-METHOXYFLAVONE	C16H12O3	252.27	4143-74-2	flavone		*Sapindus saponaria.*
435	538977971	01504002	BAICALEIN	C15H10O5	270.24	491-67-8	flavone	antiviral (HIV)	*Scutellaria baicalensis*
436	538977927	01500672	QUERCETIN	C15H10O7	302.24	117-39-5	flavone	capillary protectant, antioxidant. antineoplastic, anti-HIV; LD50(mouse) 159 mg/kg po	*Solanaceae, Rhamnaceae, Passifloraceae, Umbelliferae genera*
437	538977555	00300538	QUERCETIN TETRAMETHYL (5,7,3′,4′) ETHER	C19H18O7	358.35		flavone		*Sterculia foetida*
438	538987999	01505144	5,7-DIHYDROXYFLAVONE	C15H10O4	254.24	480-40-0	flavone		*Ulnus sieboldiana, Flourensia resinosa, Oroxylum indicum, Pinus and Scutellaria* spp.
439	538978134	01504068	DIOSMETIN	C16H12O6	300.27	520-34-3	flavone		*Valeriana, Digitalis* spp.
440	538986002	01500724	GENKWANIN	C16H12O5	284.27	437-64-9	flavone		*widespread in plants*
441	538977972	01504003	CENTAUREIN	C24H26O13	522.47	35595-03-0	flavone glycoside		*Centaurea jacea*
442	538978136	01504075	RHOIFOLIN	C27H30O14	578.53	17306-46-6	flavone glycoside		*Chorisia, Citrus and Rhus* spp.
443	1201579	00200115	PECTOLINARIN	C29H34O15	622.59	28978-02-1	flavone glycoside		*Cirsium and Linaria* spp., *Kickxia elatine, Duranta plumieri*
444	538977948	00310011	HESPERIDIN	C28H34O15	610.57	520-26-3	flavone glycoside	capillary protectant	*Citrus* spp.
445	538978102	00211950	COSMOSIIN	C21H20O10	432.39	578-74-5	flavone glycoside	antiviral (HIV)	*Cosmos bipinnatus, Zinnia elegans*
446	538986631	01505257	ICARIIN	C33H40O15	676.68	489-32-7	flavone glycoside	hepatoprotective	*Epimedium* spp.
447	538987982	01505127	GOSSYPIN	C21H20O13	480.39	652-78-8	flavone glycoside		*Gossypium* spp., *Hibiscus* spp.
448	538978101	00350025	APIIN	C26H28O14	564.50	26544-34-3	flavone glycoside		parsley seed; flowers of Anthemis nobilis. 80% + other glycosides
449	538986571	00300607	RUTOSIDE (rutin)	C27H30O16	610.53	153-18-4	flavone glycoside	vascular protectant	Ruta graveolens, widespread in plants
450	538977895	01500752	QUERCITRIN	C21H20O11	448.39	522-12-3	flavone	antihemmorrhagic	widespread in plants

							glycoside		
451	505	01504029	LIGUSTILIDE	C12H14O2	190.24	4431-01-0	furan	antispasmodic, smooth muscle relaxant	*Ligusticum and Angelica* spp.
452	538986029	00200046	GRISEOFULVIN	C17H17ClO6	352.77	126-07-8	grisan	antifungal, inhibits mitosis in metaphase	*Penicillium griseofulvum*
453	1210773	00200243	GRISEOFULVIC ACID	C16H15ClO6	338.75	469-54-5	grisan		semisynthetic
454	538988006	01505151	HARPAGOSIDE	C23H28O11	480.47	19210-12-9	irioid		*Melittis melissophyllum, Harpagophytum procumbens*
455	3148	00201177	DUARTIN (−)	C18H20O6	332.36	52305-04-1	isoflavan		*Dalbergia variabilis*
456	3150	00201364	DUARTIN, DIMETHYL ETHER	C20H24O6	360.41		isoflavan		derivative of DUARTIN (00201177)
457	538977540	00240565	5,7-DIHYDROXYISOFL AVONE	C15H10O4	254.24	4044-00-2	isoflavone		*Arachis hypogaea& Derris* spp.
458	538986784	00200422	KOPARIN	C16H12O6	300.27	65048-75-1	isoflavone		*Castanospermum australe*
459	538986430	00240576	5,7-DIMETHOXYISOFL AVONE	C17H14O4	282.30	26964-35-2	isoflavone		*Cordyla africana, Arachis hypogaea*
460	538986282	00200139	ISOTECTORIGENIN, 7-METHYL ETHER	C18H16O6	328.32		isoflavone		*Dalbergia* spp.
461	538987886	01700330	MUNDULONE ACETATE	C28H28O7	476.53		isoflavone		derivative
462	538977590	00200873	IRIGENIN TRIMETHYL ETHER	C21H22O8	402.40		isoflavone		derivative
463	538986416	00240645	RETUSIN 7-METHYL ETHER	C17H14O5	298.30		isoflavone		derivative Dalbergia spp.
464	538976321	10100004	BIOCHANIN A, 7-METHYL ETHER	C17H14O5	298.30	34086-51-6	isoflavone		derivative of biochanin A
465	538976313	01501208	KARANJIN	C17H10O4	278.27	521-88-0	isoflavone		*Derris and Tephrosia* spp.
466	3594	00201650	DERRUBONE	C21H18O6	366.37	22044-58-2	isoflavone		*Derris robusta*
467	538986653	01401419	DERRUSTONE	C18H14O6	326.31	2204-59-3	isoflavone		*Derris robusta*
468	1207050	00210454	ROBUSTONE	C21H16O6	364.36		isoflavone		*Derris robusta, Millettia thonningii*
469	538977785	01401401	METHYL ROBUSTONE	C22H18O6	378.39		isoflavone		*Derris* spp.
470	3205	00201310	2′-METHOXYFORMON ETIN	C17H14O5	298.30		isoflavone		*Eschscholtzia californica*
471	538976392	00300601	BIOCHANIN A DIACETATE	C20H16O7	368.35		isoflavone		Fabaceae; biochanin derivative
472	538977580	00201182	IRIGENOL	C15H10O8	318.24	4935-93-7	isoflavone		*Iris* spp.

473	21280	01504166	ISOOSAJIN	C25H24O5	404.47	5745-54-0	isoflavone		*Maclura pomifera*
474	3602	00201580	POMIFERIN	C25H24O6	420.47	572-03-2	isoflavone	antioxidant	*Maclura pomifera*
475	3613	00201595	OSAJIN	C25H24O5	404.47	482-53-1	isoflavone		*Maclura pomifera*
476	1205721	00200011	MUNDULONE	C26H26O6	434.49	481-94-7	isoflavone		*Mundulea sericea*
477	538986415	00200416	KOPARIN 2′-METHYL ETHER	C17H14O6	314.30		isoflavone		*Myroxylon peruiferum*
478	538986685	01505282	BIOCHANIN A, DIMETHYL ETHER	C18H16O5	312.33	1162-82-9	isoflavone		*Ouratea hexasperma*
479	3465	00201341	ISOTECTORIGENIN TRIMETHYL ETHER	C19H18O6	342.35		isoflavone		parent *Dalbergia* spp., Millettia auriculata
480	1919	00102018	ICHTHYNONE	C23H20O7	408.41	24340-62-3	isoflavone	piscicide	*Piscidia erythrina and Millettia rubiginosa*
481	538977601	00200789	DAIDZEIN	C15H10O4	254.24	486-66-8	isoflavone	phytoestrogen	red clover
482	538977502	00102007	FORMONONETIN	C16H12O4	268.27	485-72-3	isoflavone	phytoestrogen	soyabean and clover species
483	538976320	10100003	BIOCHANIN A	C16H12O5	284.27	491-80-5	isoflavone	phytoestrogen	widely distributed in Leguminosae
484	2381	00210296	GENISTEIN	C15H10O5	270.24	446-72-0	isoflavone		widely distributed in Leguminoseae
485	538977659	00201328	TECTORIGENIN	C16H12O6	300.27		isoflavone		widespread in Fabaceae
486	538977589	00200793	IRIDIN	C24H26O13	522.47	491-74-7	isoflavone glycoside		*Iris* spp.
487	538986411	01504411	PICROPODOPHYLLOT OXIN ACETATE	C24H24O9	456.45		lignan		derivative
488	538986412	01504412	PODOPHYLLOTOXIN ACETATE	C24H24O9	456.45	1180-34-3	lignan		derivative
489	538986437	01504739	beta-PELTATIN	C22H22O8	414.42	518-29-6	lignan	antineoplastic, cytotoxic	*Podophyllum* spp.
490	538977381	02300332	PODOFILOX	C22H22O8	414.42	518-28-5	lignan	antineoplastic, inhibits microtubule assembly, and human DNA topoisomerase II; antimitotic agent	*Podophylum peltatum; podophylotoxin*
491	538986410	01504410	PICROPODOPHYLLOT OXIN	C22H22O8	414.42	477-47-4	lignan	antineoplastic; 10% cytotoxicity of podophyllotoxin	Podophylum peltatum; epimer of podophyllotoxin; 10% mitotoxicity
492	5	00100005	ANTHOTHECOL	C28H32O7	480.56	10410-83-0	limonoid		also as 11-ACETOXYCEDREL ONE

493	538978141	00100102	CARAPIN-8(9)-ENE	C27H30O7	466.54		limonoid		Carapa and Cedrela species
494	1201054	00100008	CARAPIN	C27H32O7	468.55	3463-88-5	limonoid		Carapa and Cedrela species; mp 180–185
495	2	00100003	ANDIROBIN	C27H32O7	468.55	6488-63-7	limonoid		*Carapa guayanensis*
496	538976329	00100031	FISSINOLIDE	C29H36O8	512.61	1915-69-1	limonoid		*Cedrela fissilis, Khaya grandifoliola*
497	1201055	00100009	CEDRELONE	C26H30O5	422.53	1254-85-9	limonoid		Cedrela species
498	538986760	01505341	NOMILIN	C28H34O9	514.58	1063-77-0	limonoid	antineoplastic	*Citrus* spp.
499	1204174	01800018	LIMONIN	C26H30O8	470.52	1180-71-8	limonoid		*Citrus, Evodia, Dictamnus and Luvunga* spp.
500	538978077	00100199	DEOXYGEDUNOL ACETATE	C30H38O7	510.63		limonoid		derivative
501	538978142	00100105	8beta-HYDROXYCARAPIN, 3,8-HEMIACETAL	C27H32O8	484.55		limonoid		derivative
502	1201236	00100655	DIHYDROGEDUNIC ACID, METHYL ESTER	C26H36O8	476.57		limonoid		derivative
503	1201231	00100375	alpha-DIHYDROGEDUNOL	C28H38O7	486.61		limonoid		derivative
504	1201549	00100359	3,16-DIDEOXYMEXICANOLIDE-3beta-DIOL	C27H36O7	472.58		limonoid		derivative
505	1201545	00100222	3-DEOXO-3beta-HYDROXYMEXICANOLIDE 16-ENOL ETHER	C28H36O7	484.59		limonoid		derivative
506	538976330	00100465	DIHYDROFISSINOLIDE	C29H38O8	514.62		limonoid		derivative of fissinolide (00100031)
507	1210776	00100146	7-DESACETOXY-6,7-DEHYDROGEDUNIN	C26H30O5	422.53		limonoid		derivative of gedunin
508	538976339	00100054	ANGOLENSIC ACID, METHYL ESTER	C27H34O7	470.57	2629-14-3	limonoid		Entandrophragma angolense
509	6	00100006	BUSSEIN	C43H54O18	858.90	41060-14-4	limonoid		Entandrophragma species
510	1201695	00100081	UTILIN	C41H52O17	816.86	31218-22-1	limonoid		Entandrophragma utile
511	538985967	00100517	ENTANDROPHRAGMIN	C43H56O17	844.92	11013-05-1	limonoid		Entangrophragma spp.
512	1201459	00100103	KHAYASIN C	C30H38O8	526.63		limonoid		ester analog of Khayasin (00100050)
513	1201375	00100195	ISOGEDUNIN	C28H34O7	482.58		limonoid		Guarea Thompsonii; mp 215–218
514	1201462	00100162	KHIVORIN	C32H42O10	586.69	2524-38-1	limonoid		Khaya and other West

									African timbers
515	3	00100016	DEOXYANDIROBIN	C27H32O6	452.55		limonoid		Khaya grandifoliola
516	1201453	00100002	11alpha-ACETOXYKHI VORIN	C34H44O12	644.72		limonoid		Khaya madagascariensis
517	1201455	00100048	7-DEACETOXY-7-OXO KHIVORIN	C30H38O9	542.63	15004-51-0	limonoid		Khaya senegalensis and other Meliaceae; mp 225
518	1201457	00100050	KHAYASIN	C32H42O8	554.69		limonoid		Khaya species
519	1201456	00100049	KHAYANTHONE	C32H42O9	570.69	25279-68-9	limonoid		Khaya species
520	1205624	00100014	7-DEACETYLKHIVORI N	C30H40O9	544.65		limonoid		Khaya species and other West African timbers
521	538977839	00100576	TRIDESACETOXYKHIV ORIN	C26H36O7	460.57		limonoid		Khaya spp.
522	1201293	00100012	DEACETYLGEDUNIN	C26H32O6	440.54		limonoid		Khaya, Azadirachta and other West African timbers; mp 264–266
523	538985526	01503802	AZADIRACHTIN	C35H44O16	720.73	11141-17-6	limonoid	antifeedant, insecticide	*Melia azadirach and Azadirachta indica*
524	1201073	00100615	CHUKRASIN METHYL ETHER	C43H58O16	830.93		limonoid		Meliaceae
525	538986667	00212126	1,3-DIDEACETYLDEOX YKHIVORIN	C28H38O7	486.61		limonoid		*Meliaceae* spp.
526	538986086	00100901	3beta-HYDROXYDEOX YDESACETOXY-7-OXO GEDUNIN	C26H32O6	440.54		limonoid		*Meliaceae* spp.
527	538978179	00100290	2,3-DIHYDROISOGEDU NIN	C28H36O7	484.59		limonoid		*Meliaceae* spp.
528	1201394	00100434	DIHYDRODEOXYGED UNIN	C28H36O6	468.60		limonoid		*Meliaceae* spp.
529	1205418	00100439	1(2)alpha-EPOXYDEOX YDIHYDROGEDUNIN	C28H34O7	482.58		limonoid		*Meliaceae* spp.
530	538978062	00100056	6-ACETOXYANGOLEN SIC ACID METHYL ESTER	C29H36O9	528.60		limonoid		*Meliaceae* spp.
531	1201146	00100368	1,2alpha-EPOXY-7-DEA CETOXY-7-OXODIHYD ROGEDUNIN	C26H30O7	454.52		limonoid		*Meliaceae* spp.
532	538986088	00102034	3beta,7beta-DIACETOX YDEOXODEACETOXY DEOXYDIHYDROGED	C30H40O7	512.65		limonoid		*Meliaceae* spp.

			UNIN					
533	538978205	00100423	DEACETOXY-7-OXISO GEDUNIN	C26H30O6	438.53		limonoid	*Meliaceae* spp.
534	1201711	00100424	XYLOCARPUS A	C31H38O11	586.64		limonoid	*Meliaceae* spp.
535	1210759	00100013	3-DEACETYLKHIVORI N	C30H40O9	544.65		limonoid	*Meliaceae* spp.
536	538985916	00100190	3-alpha-HYDROXYDEO XYGEDININ	C28H36O6	468.60		limonoid	*Meliaceae* spp.
537	1201480	00100585	1,7-DIDEACETOXY-1,7-DIOXOKHIVORIN	C28H34O8	498.58		limonoid	*Meliaceae* spp.
538	538986296	00300558	GEDUNOL	C28H36O7	484.59		limonoid	*Meliaceae* spp.
539	1201134	00100201	3-DEOXO-3beta-ACETO XYDEOXYDIHYDROGE DUNIN	C30H40O7	512.65		limonoid	*Meliaceae* spp.
540	538986044	00100651	3beta-ACETOXYDEOXY ANGOLENSIC ACID, METHYL ESTER	C29H38O8	514.62		limonoid	*Meliaceae* spp.
541	538986087	00102035	3beta-HYDROXYDEOX ODIHYDRODEOXYGE DUNIN	C28H38O6	470.61		limonoid	*Meliaceae* spp.
542	1201465	00100060	MEXICANOLIDE	C27H32O7	468.55	1915-67-9	limonoid	*Meliaceae* spp.
543	1201321	00100432	DEOXYGEDUNIN	C28H34O6	466.58		limonoid	*Meliaceae* spp.
544	538985928	00100205	3alpha-ACETOXYDIHY DRODEOXYGEDUNIN	C30H40O7	512.65		limonoid	*Meliaceae* spp.
545	538977632	00100047	DEACETOXY-7-OXOGE DUNIN	C26H30O6	438.53		limonoid	*Meliaceae* spp.
546	538978125	00100173	EPOXYGEDUNIN	C28H34O8	498.58		limonoid	*Meliaceae* spp.
547	538985929	00100358	1,7-DIDEACETOXY-1,7-DIOXO-3-DEACETYLK HIVORIN	C26H32O7	456.54		limonoid	*Meliaceae* spp.
548	538985940	00100139	DEOXYKHIVORIN	C32H42O9	570.69		limonoid	*Meliaceae* spp.
549	538978068	00100129	8-HYDROXYCARAPINI C ACID	C26H30O8	470.52		limonoid	*Meliaceae* spp.
550	538978063	00100058	6-HYDROXYANGOLE NSIC ACID METHYL ESTER	C27H34O8	486.57		limonoid	*Meliaceae* spp.
551	1201227	00100492	beta-DIHYDROGEDUN OL	C28H38O7	486.61		limonoid	*Meliaceae* spp.
552	538978067	00100114	3alpha-HYDROXY-3-DE OXYANGOLENSIC ACID METHYL ESTER	C27H36O7	472.58		limonoid	*Meliaceae* spp.
553	538978065	00100096	3-DEOXY-3beta-HYDR	C27H36O7	472.58		limonoid	*Meliaceae* spp.

			OXYANGOLENSIC ACID METHYL ESTER						
554	1201228	00100497	3beta-ACETOXYDEOXODIHYDROGEDUNIN	C30H40O8	528.65		limonoid		*Meliaceae* spp.
555	1201136	00100223	1,2alpha-EPOXYDEACETOXYDIHYDROGEDUNIN	C26H32O7	456.54		limonoid		*Meliaceae* spp.
556	1201467	00100355	1,3-DIDEACETYL-7-DEACETOXY-7-OXOKHIVORIN	C26H34O7	458.56		limonoid		*Meliaceae* spp.
557	538976347	00100024	DIHYDROGEDUNIN	C28H36O7	484.59		limonoid		*Meliaceae* spp.
558	1201296	00100032	GEDUNIN	C28H34O7	482.58	2753-30-2	limonoid	antifeedant; heat shock inducer	numerous Meliaceae species
559	538977915	00240930	AVOCADANE ACETATE	C19H38O4	330.51		lipid		avocado seeds
560	538977914	00240560	AVOCADENE ACETATE	C19H36O4	328.50	24607-09-8	lipid	antifungal, plant growth inhibitor	avocado seeds
561	538977847	10101011	BIXIN	C25H30O4	394.52	39937-23-0	lipid		Bixa orellana seeds
562	538986658	01505276	CAPSANTHIN	C40H56O3	584.89	465-42-9	lipid	antineoplastic	Capsicum annum
563	1119	01501128	CAPSAICIN	C18H27NO3	305.42	404-86-4	lipid	analgesic (topical), depletes Substance P, neurotoxic	Capsicum spp.
564	538977780	01503674	ANTHERAXANTHIN	C40H56O3	584.89	640-03-9	lipid		Euonymus europaeus
565	1210908	01501203	RETINOL	C20H30O	286.46	68-26-8	lipid	vitamin A	fish & liver oils, eggs, milk
566	538977957	01503983	BATYL ALCOHOL	C21H44O3	344.58	544-62-7	lipid		fish oils
567	538987929	01505247	GINKGOLIC ACID	C22H34O3	346.51	22910-60-7	lipid	antibacterial, antitubercular	Ginkgo biloba
568	538977621	00310016	CHAULMOOGRIC ACID	C18H32O2	280.45	502-30-7	lipid	antibacterial (mycobacteria)	*Hydnocarpus and Oncola* spp.
569	538976350	00200428	ROCCELLIC ACID	C17H32O4	300.44	22139-54-4	lipid		*Lecanora and Roccella* spp.
570	538987905	00240928	DIMETHYL CAPERATATE	C23H42O7	430.59		lipid		*lichens: Parmelia, Mycoblastus, Nephromopsis* spp.
571	538987904	00240927	CAPERATIC ACID	C21H38O7	402.53	29227-64-3	lipid	antibacterial (tuberculostatic)	*lichens: Parmelia, Mycoblastus, Nephromopsis* spp.
572	538977626	00310021	PHYTOL	C19H38O	282.51		lipid		nettles
573	538987892	01505325	SOLANESOL	C45H74O	631.09	13190-97-1	lipid		Nicotiana tabacum; Betulaprenol 9: mp 42 C

574	538987893	01505326	SOLANESYL ACETATE	C47H76O2	673.13		lipid		Nicotiana tabacum; *Murraya exotica; Pinus* spp.
575	538987889	01505807	AVOCADENOFURAN	C17H28O	248.41	25346-24-1	lipid		*Persea* spp.
576	538986646	01505234	AVOCADYNE	C17H32O3	284.44	34524-38-4	lipid	antibacterial, antifungal	*Persea* spp.
577	538987888	01505806	AVOCADANOFURAN	C17H30O	250.43		lipid		*Persea* spp.
578	538978239	00240929	AVOCADYNE ACETATE	C19H34O4	326.48	24607-06-5	lipid	antifungal	*Persea* spp.
579	538987890	01505808	AVOCADYNOFURAN	C17H26O	246.40	24708-33-6	lipid		*Persea* spp. *and Elodia canadensis*
580	538987877	00240932	AVOCADENE	C17H34O3	286.46	24607-08-7	lipid	antifungal	*Persia americana*; avocado seeds
581	538977644	00310039	alpha-TOCHOPHEROL	C29H50O2	430.72	59-02-9	lipid	vitamin E	soya, wheat germ and other plant oils
582	1204093	01800172	BYSSOCHLAMIC ACID	C18H20O6	332.36	743-51-1	macrolide		*Byssochlamys* spp.
583	1201115	00300037	CRASSIN ACETATE	C22H32O5	376.50	28068-69-1	macrolide	antiviral	numerous gorgonids; mp 123–125 C
584	538986417	00700024	HAEMATOPORPHYRIN DIHYDROCHLORIDE	C34H40Cl2N4O6	671.63	17696-69-4	porphyrin	antidepressant, antineoplastic	*Chlorella vulgaris*; derived blood
585	1210772	01501111	PROTOPORPHYRIN IX	C34H34N4O4	562.67	553-12-8	porphyrin	hepatoprotectant	mamalian feces, avian pigment
586	13	01500857	BILIRUBIN	C33H36N4O6	584.68	635-65-4	porphyrin		pigment mamalian gallstones, blood and urine
587	538985487	01503904	PATULIN	C7H6O4	154.12	149-29-1	pyran	antibacterial	*Aspergillus clavatus, Penicillium patulum*
588	1204168	01800166	KOJIC ACID	C6H6O4	142.11	501-30-4	pyran	chelator, antibacterial, skin whitening agent	*Aspergillus* spp.
589	538977630	00310025	LARIXINIC ACID	C6H6O3	126.11	118-71-8	pyran		*Larix decidua*
590	538986119	01600759	4-HYDROXY-6-METHYLPYRAN-2-ONE	C6H6O3	126.11	675-10-5	pyran		*Pennicillium stipitatum*
591	538985690	01504090	2-PROPYL-3-HYDROXYETHYLENEPYRAN-4-ONE	C10H12O3	180.21		pyran		Withania somnifera
592	1201472	00100455	MEROGEDUNIN	C21H28O4	344.45		quassinoid		derivative
593	538977634	00310028	QUASSIN	C22H28O6	388.46	76-78-8	quassinoid	insecticide, antiamoebic	*Quassia amara, Picrasma excelsa and Ailanthus glandulosa*
594	538985665	01500223	DAUNORUBICIN	C27H29NO10	527.53	20830-81-3	quinone	antineoplastic	*Streptomyces peucetius;*

									FI-6339, NDC-0082-4155, RP-13057
595	538977505	00300556	CHRYSAROBIN	C15H12O3	240.26	491-58-7	quinone		*Andira araroba* (glacial AcOH)
596	538987984	01505129	PLUMBAGIN	C11H8O3	188.18	481-42-5	quinone	antibacterial, antifungal, tuberculostatic; antifeedant (African army worms)	*Aristea, Diospyros & Plumbago* spp.; *Dyerophyton, Drosera, Dioncophyllum, Nepenthe, Sisyrinchium and Sparaxis tricolor*
597	538985669	01502254	MENADIONE	C11H8O2	172.19	58-27-5	quinone	prothrombogenic agent	*Asplenium and Juglans* spp.
598	538977654	00310299	THYMOQUINONE	C10H12O2	164.21		quinone		*Callitris, Monarda* spp., *Juniperus cedrus, Nigella sativa*
599	1210714	01500898	EMODIN	C15H10O5	270.24	518-82-1	quinone	antibacterial, antineoplastic, cathartic, tyrosine kinase inhibitor	Cascara, Rheum and Rhammnus species
600	860	00201664	CELASTROL	C29H38O4	450.62	34157-83-0	quinone	antineoplastic, antiinflamatory, NO synthesis inhibitor, chaperone stimulant	*Celastrus scandens & Tripterygium wilfordii*
601	2990	00201448	4,4′-DIMETHOXYDALBERGIONE	C17H16O4	284.31		quinone		*Dalbergia nigra*
602	1201661	00200255	RETUSOQUINONE	C11H12O	160.22		quinone		*Dalbergia retusa*
603	2991	00201092	4-METHOXYDALBERGIONE	C16H14O3	254.29	4646-86-0	quinone		*Dalbergia retusa and D. nigra*
604	538977596	00201281	DALBERGIONE	C15H12O2	224.26		quinone		*Dalbergia* spp.
605	2992	00200798	DALBERGIONE, 4-METHOXY-4′-HYDROXY-	C16H14O4	270.29		quinone		*Dalbergia* spp.
606	538977721	00240828	3,4-DIMETHOXYDALBERGIONE	C17H16O4	284.31	41043-20-3	quinone	induces dermatitis	*Dalbergia* spp., *Machaerium* spp.
607	538978135	01504074	EMBELIN	C17H26O4	294.39		quinone	anthelmintic, oral contraceptive	*Embelia* spp.
608	538976309	01501204	LAPACHOL	C15H14O3	242.28	84-79-7	quinone	antineoplastic, antifungal	heartwood of *Bignoniaceae*
609	538976410	01502250	LAWSONE	C10H6O3	174.16	83-72-7	quinone		*Lawsonia* spp.
610	1201448	00300038	JUGLONE	C10H6O3	174.16	481-39-0	quinone	antineoplastic, antifungal	leaves and nuts of *Juglans* spp., *Carya* spp.

									andPterocarya spp.
611	538986422	00200413	2,6-DIMETHOXYQUINONE	C8H8O4	168.15	35069-70-6	quinone	antibacterial, induces dermatitis, mutagen	*Picrasmus & Ailanthus* spp.
612	135	00211468	DANTRON	C14H8O4	240.22	117-10-2	quinone	cathartic	*Rheum palmatum, Xyris semifuscata*
613	538987921	01505812	CRYPTOTANSHINONE	C19H20O3	296.37	35825-57-1	quinone	inhibits angiogenesis	*Salvia miltiorrhiza, Rosmarinus officinalis*
614	538987918	01505825	DIHYDROTANSHINONE I	C18H14O3	278.31		quinone		*Salvia miltiorrhiza*
615	538987917	01505824	TANSHINONE IIA	C19H18O3	294.35	568-72-9	quinone	antineoplastic, bone resorption inhibitor, antiproliferative, apoptosis inducer	*Salvia miltiorrhiza*
616	538977583	00200463	BRAZILEIN	C16H12O5	284.27	600-76-0	quinone methide		*Caesalpinia* spp.
617	1201578	00200090	OBTUSAQUINONE	C16H14O3	254.29	21105-15-7	quinone methide		*Dalbergia retusa*
618	538977922	00210186	CITRININ	C13H14O5	250.25	518-75-2	quinone methide	antibacterial	*Penicillium citrinum*
619	538977764	00202175	12a-HYDROXY-5-DEOXYDEHYDROMUNDUSERONE	C19H18O6	342.35		rotenoid		derivative
620	538977759	00200004	12a-HYDROXY-9-DEMETHYLMUNDUSERONE-8-CARBOXYLIC ACID	C19H16O9	388.33		rotenoid		derivative
621	1201674	00200851	ROTENONIC ACID	C23H24O6	396.44	70191-71-8	rotenoid		derivative of rotenone (00200013)
622	3443	00201449	DIHYDROROTENONE	C23H24O6	396.44		rotenoid		derivative; mp 209–211 C
623	3781	00203010	beta-TOXICAROL	C23H22O7	410.43	82-11-1	rotenoid		Derris species & isomeration of alpha-toxicarol
624	2746	00211224	alpha-TOXICAROL	C23H22O7	410.43	82-09-7	rotenoid		*Derris* spp.
625	428	00200013	ROTENONE	C23H22O6	394.43	83-79-4	rotenoid	acaricide, ectoparasiticide, antineoplastic, mitochondrial poison	*Derris* spp.
626	3442	00201153	beta-DIHYDROROTENONE	C23H24O6	396.44		rotenoid		*Derris* spp.; rotenone derivative
627	3441	00201154	DEHYDROROTENONE	C23H20O6	392.41	30990-44-4	rotenoid		*Derris* spp.; rotenone

									derivative
628	3567	00201477	MUNDOSERONE	C19H18O6	342.35	3564-85-0	rotenoid		Mundulea suberosa; mp 156–161 C
629	538977682	00201657	PACHYRRHIZONE	C20H14O7	366.33	42485-00-7	rotenoid	insecticide	*Pachyrrhizus erosus*
630	1201420	00200015	ISOROTENONE	C23H22O6	394.43		rotenoid		semisynthetic
631	2993	00201138	DEGUELIN(−)	C23H22O6	394.43	522-17-8	rotenoid	antineoplastic, antiviral, insecticide	*Tephrosia & Derris* spp.
632	538976382	00307057	BISABOLOL	C15H26O	222.37	515-69-5	sesquiterpene	antiinflammatory, antiulcer, antiseptic, antitubercular	*Abies, Pinaceae* and related species
633	67	01501107	PICROTOXININ	C15H16O6	292.29	17617-45-7	sesquiterpene	convulsant, GABA receptor antagonist, ichthyotoxin	*Anamirta cocculus, Menispermum cocculus*
634	538986059	01504149	LEUCODIN	C15H18O3	246.31	17946-87-1	sesquiterpene	antiinflammatory, hypolipidemic	*Artemesia & Achillea* spp.
635	538977448	01503042	ARTEMISININ	C15H22O5	282.34	63968-64-9	sesquiterpene	antimalarial	*Artemisia annua*
636	538977506	00300542	SANTONIN	C15H18O3	246.31	481-06-1	sesquiterpene		*Artemisia* spp.
637	1205340	00300055	CADIN-4-EN-10-OL	C15H26O	222.37		sesquiterpene		*Chamaecyparis* spp. *and Juniperus* spp.
638	1210719	01503640	PARTHENOLIDE	C15H20O3	248.32	20554-84-1	sesquiterpene	5HT antagonist, antineoplastic, smooth muscle relaxant	*Chrysanthemum parthenium, Michelia champaca*
639	1210699	01500842	CARYOPHYLLENE [t(−)]	C14H22	190.33	87-44-5	sesquiterpene		clove, cinnamon and many other oils
640	1210690	01500832	CARYLOPHYLLENE OXIDE	C14H22O	206.33	1139-30-6	sesquiterpene		clove, cinnamon and many other oils
641	538976381	00307059	CEDROL	C15H26O	222.37	77-53-2	sesquiterpene	acaricide	Common constitutent in the Family Cupressaceae
642	538976411	01502251	NEROLIDOL	C15H26O	222.37	7212-44-4	sesquiterpene		common in essential oils of flowers
643	538986049	01504139	AUSTRICINE	C15H18O4	262.31	10180-88-8	sesquiterpene	antihyperlipidemic, antiinflammatory	Compositae
644	1205968	00300048	KOBUSONE	C14H22O2	222.33	24173-71-5	sesquiterpene		*Cyperus rotundus, Sindora sumatrana*
645	1204849	00300117	ISOKOBUSONE	C14H22O2	222.33	24173-72-6	sesquiterpene		*Cyperus rotundus, Sindora sumatrana*
646	1201275	00300049	5-HYDROXYIMINOISO CARYOPHYLLENE	C15H23NO	233.36		sesquiterpene		derivative
647	538976383	00307058	BISABOLOL ACETATE	C17H28O2	264.41		sesquiterpene		derivative
648	1204972	00300119	2-HYDROXY-5	C15H26O2	238.37		sesquiterpene		derivative

			(6)EPOXY-TETRAHYD ROCARYOPHYLLENE						
649	1204566	00300104	2-METHYLENE-5-(2,5-D IOXOTETRAHYDROFU RAN-3-YL)-6-OXO--10,1 0-DIMETHYLBICYCLO[7: 2: 0]UNDECANE	C18H24O4	304.39		sesquiterpene		derivative
650	1204973	00300111	2-METHOXY-5 (6)EPOXY-TETRAHYD ROCARYOPHYLLENE	C16H28O2	252.40		sesquiterpene		derivative
651	538977913	00300166	15-NORCARYOPHYLL EN-3-ONE	C14H22O	206.33		sesquiterpene		derivative
652	1201869	00300133	CLOVANEDIOL DIACETATE	C19H30O4	322.45		sesquiterpene		*derivative Dipterocarpus pilosus, Salvia canariensis, Viguiera oaxacana, Sindora sumatrana*
653	1201868	00300132	3,7-EPOXYCARYOPHY LLA*N*-6-ONE	C15H24O2	236.36		sesquiterpene		derivative *Lippia* spp.
654	1204974	00300118	3,7-EPOXYCARYOPHY LLA*N*-6-OL	C15H26O2	238.37		sesquiterpene		derivative *Lippia* spp.
655	538976386	00307056	MUUROLLADIE-3-ON E	C15H22O	218.34		sesquiterpene		derivative of muurolene
656	1205010	00300110	3-NOR-3-OXOPANASI NSAN-6-OL	C14H22O2	222.33		sesquiterpene		derivative Panax ginseng
657	1201758	00300052	SENECRASSIDIOL 6-ACETATE	C17H28O3	280.41		sesquiterpene		derivative Senecio crassissimus
658	538977678	00300148	CARYOPHYLLENYL ACETATE	C17H26O2	262.40		sesquiterpene		*Dipterocarpus* spp.
659	538987937	01505186	CULMORIN	C15H26O2	238.37	18374-83-9	sesquiterpene		*Fusarium* spp.
660	7	01501210	HUMULENE (alpha)	C15H24	204.36	6753-98-6	sesquiterpene		hops and clove oils
661	1204619	00300101	EPOXY (1,11)HUMULENE	C15H24O	220.36		sesquiterpene		Humulus lupulus
662	538976364	00240914	PSEUDO-ANISATIN	C15H22O6	298.34	31090-37-6	sesquiterpene	GABA antagonist	Illicium anisatum
663	538980369	00310010	HELENINE	C15H20O2	232.33	546-43-0	sesquiterpene	anthelmintic, antibacterial, antineoplastic	Inula spp.
664	19	01501022	FARNESOL	C15H26O	222.37	4602-84-0	sesquiterpene		major component in oil of *Hibiscus abelmoschus*
665	538980373	00100346	PICROTIN	C15H18O7	310.31	21416-53-5	sesquiterpene	GABAa receptor antagonist	nontoxin component of PICROTOXIN

666	1204710	00300105	beta-CARYOPHYLLEN E ALCOHOL	C15H26O	222.37		sesquiterpene		*palmarosa oil, Cymbopogon martini*
667	1204135	01800170	FREQUENTIN	C14H20O4	252.31	29119-03-7	sesquiterpene	antifungal	*Penicillium frequentans*
668	818	00400011	PUNCTAPORONIN B	C15H24O3	252.36	93697-36-0	sesquiterpene		*Poronia punctata*
669	538977620	00310015	CEDRYL ACETATE	C17H28O2	264.41	77-54-3	sesquiterpene		semisynthetic
670	538986054	01504143	ARTENIMOL	C15H24O5	284.36	81496-81-3	sesquiterpene	antimalarial, antiinflammatory	semisynthetic; dihydroartemisinin
671	538985480	01502249	GUAIAZULENE	C15H18	198.31	489-84-9	sesquiterpene	antioxidant, inhibits lipid peroxidation inhibitor, antiinflammatory, hepatoprotectant; LD50(rat) 1550 mg/kg po	synthetic
672	538976371	01502234	ABSCISIC ACID (cis,trans; +/−)	C15H20O4	264.32		sesquiterpene	abscission-acceleran t	synthetic
673	538978190	01500835	URSOCHOLANIC ACID	C24H40O2	360.59	546-18-9	sterol		Abrus pecatorius
674	1210720	01500904	CHOLIC ACID, METHYL ESTER	C25H42O5	422.61	1448-36-8	sterol		acid as primary bile constituent
675	538986082	01701028	CORTISONE	C21H28O5	360.45	53-06-5	sterol	antiinflammatory, glucocorticoid	adrenal cortical hormone
676	538978242	00100315	TIGOGENIN	C27H44O3	416.65	77-60-1	sterol		*Agavaceae, Dioscoreaceae, Solanaceae, Scrophulariaceae, Liliaceae genera*
677	277	00100308	ROCKOGENIN	C27H44O4	432.65	16653-52-4	sterol		*Agave americana, A. gracilipes*
678	538977939	01500760	HECOGENIN	C27H42O4	430.63	467-55-0	sterol	antiinflammatory	*Agave and Yucca* spp.
679	264	00100688	DIGOXIGENIN	C23H34O5	390.52	1672-46-4	sterol		aglycon of digitoxin, thevetin, cerberin, echujin, evomonoside; mp 217–218
680	449	00100584	GITOXIGENIN DIACETATE	C27H38O7	474.60	5996-03-2	sterol		aglycon of GITOXIN
681	538977986	01500849	CHOLEST-5-EN-3-ONE	C27H44O	384.65	601-54-7	sterol		animal fats; mp 79–80 C
682	538986448	00307031	CHOLESTERYL ACETATE	C29H48O2	428.70	604-35-3	sterol		animal tissue; derivative
683	1210700	01500843	LATHOSTEROL	C27H46O	386.67		sterol		Austeria rubens
684	496	01500605	URSODIOL	C24H40O4	392.58	128-13-2	sterol	anticholelithogenic;	bear bile

								LD50(rat) 890 mg/kg ip	
685	1210718	00100566	DEOXYCHOLIC ACID	$C_{24}H_{40}O_4$	392.58	88-44-3	sterol		bile constituent
686	538978184	01504053	FUCOSTANOL	$C_{29}H_{52}O$	416.74	83-45-4	sterol		*Calendula officinalis*
687	1201536	00100093	ODORATONE	$C_{30}H_{48}O_4$	472.71		sterol		Cedrela species
688	538978103	00100318	DIOSGENIN	$C_{27}H_{42}O_3$	414.63	512-04-9	sterol	antiinflammatory, estrogen; LD50(rat) 4872 mg/kg ip. LD50 (mouse) >8000 mg/kg po	*Clintonia, Dioscorea and Solanum* spp.,*Trillium erectum, Balanites aegyptiaca, Aletris farinosa*
689	538986293	01500854	7-OXOCHOLESTEROL	$C_{27}H_{44}O_2$	400.65	566-28-9	sterol		*Cliona copiosa*
690	538986441	00307035	7-OXOCHOLESTERYL ACETATE	$C_{29}H_{46}O_3$	442.69		sterol		*Cliona copiosa*
691	1210704	01500847	CHOLESTEROL	$C_{27}H_{46}O$	386.67	57-88-5	sterol		common animal sterol
692	1144	00201696	LANOSTEROL ACETATE	$C_{32}H_{52}O_2$	468.77		sterol		derivative of lanosterol
693	1058	00200744	ERGOSTEROL ACETATE	$C_{32}H_{50}O_2$	466.75	2418-45-3	sterol		derivative; mp 179–181 C
694	538978046	00100687	GITOXIGENIN	$C_{23}H_{34}O_5$	390.52	545-26-6	sterol	antineoplastic	*Digitalis* spp.
695	493	00100583	EUPHOL ACETATE	$C_{32}H_{52}O_2$	468.77		sterol		*Euphorbia* spp.
696	1210695	01500838	CHOLECALCIFEROL	$C_{27}H_{44}O$	384.65	67-97-0	sterol	vitamin D3	fish oils
697	538986341	01800109	ERGOSTA-7,22-DIEN-3-ONE	$C_{28}H_{44}O$	396.66	32507-77-0	sterol		*Fomes & Coriolis* spp.
698	1200688	01500311	FUSIDIC ACID	$C_{31}H_{48}O_6$	516.72	6990-06-3	sterol	antibacterial	*Fusidium* spp.
699	538978086	00100310	HECOGENIN ACETATE	$C_{29}H_{44}O_5$	472.67	915-35-5	sterol		*Hechtia texensis, Agave & Yucca* spp.
700	538986424	00107108	5alpha-ANDROSTAN-3, 17-DIONE	$C_{19}H_{28}O_2$	288.43		sterol	androgen	human urine & adrenal cortex
701	538977614	00310009	EPIANDROSTERONE	$C_{19}H_{30}O_2$	290.45		sterol		in normal human urine
702	538977691	00100296	NEOTIGOGENIN ACETATE	$C_{29}H_{46}O_4$	458.69		sterol		*Lycopersicon pimpimellifolium & Agave app.*
703	1210697	01500840	CHOLIC ACID	$C_{24}H_{40}O_5$	408.58	81-25-4	sterol		mammalian bile
704	1210721	01500906	LITHOCHOLIC ACID	$C_{24}H_{40}O_3$	376.58	434-13-9	sterol	LD50(mouse) 3900 mg/kg po	mammalian bile and gallstones, faecal matter
705	538978170	01504056	EPICOPROSTEROL	$C_{27}H_{48}O$	388.68	516-92-7	sterol		mammalian excretions
706	538986292	01500852	CHOLESTANONE	$C_{27}H_{46}O$	386.67	566-88-1	sterol		mammalian tissue
707	538986438	01701001	ANDROSTA-1,4-DIEN-3 ,17-DIONE	$C_{19}H_{24}O_2$	284.40	897-06-3	sterol		mammary neoplasms; cholesterol metabolite
708	538986440	00270083	5alpha-CHOLESTAN-3b	$C_{27}H_{46}O_2$	402.67		sterol		*Mandevilla pentlandiana*

			eta-OL-6-ONE						
709	538986451	01701047	TETRAHYDROCORTISONE	C21H32O5	364.49	53-05-4	sterol		microbial degradation of cortisone
710	538986447	00270088	CHOLESTAN-3-ONE	C27H46O	386.67	566-88-1	sterol		minor mammalian sterol
711	833	00100682	SARMENTOGENIN	C23H34O5	390.52		sterol		mp 270; aglucone of sarmentocymarin *Strophanthus sarmentosus*
712	538986442	00270049	alpha-HYDROXYDEOXYCHOLIC ACID	C24H40O4	392.58	83-49-8	sterol		pig bile
713	538986439	00270090	CHOLESTAN-3beta,5alpha,6beta-TRIOL	C27H48O3	420.68	1253-84-5	sterol		*Pteroeides esperi*
714	538986443	00270051	METHYL DEOXYCHOLATE	C25H42O4	406.61	3245-38-3	sterol		rabbit bile & feces
715	538986242	01504228	CRUSTECDYSONE	C27H44O7	480.65	5289-74-7	sterol	insect molting hormone	silkworm moth *Bombyx mori*& the plant *Achyranthes fauriei*
716	538978085	00100298	SMILAGENIN ACETATE	C29H46O4	458.69		sterol		*Smilax ornata, Agave* &*Yucca* spp.
717	538977737	00107013	SMILAGENIN	C27H44O3	416.65	126-18-1	sterol		*Smilax* spp.
718	538978249	01504051	STIGMASTEROL	C29H48O	412.71		sterol		soya and calabar beans; widely distributed in plant oils
719	858	00100291	STROPHANTHIDIN	C23H32O6	404.51	66-28-4	sterol	cardiotonic	*Strophanthus kombe*
720	1201535	00100090	NILOTICIN	C30H48O3	456.72		sterol		*Turraea nilotica*
721	538986452	01701060	HYDROXYPROGESTERONE	C21H30O3	330.47	3168-01-2	sterol	progestagen	urine and blood
722	538985930	00107022	beta-SITOSTEROL	C29H50O	414.72	83-46-5	sterol		widespread in plants
723	538977744	00107023	SITOSTERYL ACETATE	C31H52O2	456.76		sterol		widespread in plants
724	538976401	00200743	ERGOSTEROL	C28H44O	396.66	57-87-4	sterol		yeast
725	353	01500676	OUABAIN	C29H44O12	584.67	11018-89-6, 630-60-4 [anhydrous]	sterol glycoside	antiarrhythmic, cardiotonic, hypertensive, Na/K ATPase inhibitor	*Acokanthera* and *Strophanthus* spp.
726	265	01500247	DIGOXIN	C41H64O14	780.96	20830-75-5	sterol glycoside	cardiac stimulant	*Digitalis lanata or D. orientalis Lam., Scrophulariaceae*
727	259	00100325	DIGITONIN	C56H92O29	1229.34	11024-24-1	sterol glycoside		*Digitalis purpurea*
728	538977587	01500246	DIGITOXIN	C41H64O13	764.96	71-63-6	sterol glycoside	inotropic,	*Digitalis* spp.

								cardiotonic	
729	1210737	01500986	GITOXIN	C41H64O14	780.96	4562-36-1	sterol glycoside	cardiotonic	*Digitalis* spp.
730	538978107	01504018	HEDERACOSIDE C	C59H96O26	1221.41		sterol glycoside		Hedera helix
731	839	00100568	SARMENTOSIDE B	C34H48O13	664.75		sterol glycoside		*Strophanthus sarmentosus*; mp 193–195
732	538978124	01504044	RESVERATROL 4′-METHYL ETHER	C15H14O3	242.28	33626-08-3	stilbene		derivative
733	538978118	01504041	TRIACETYLRESVERAT ROL	C20H18O6	354.36	42206-94-0	stilbene		*Kirkpatrickia variolosa* (sponge)
734	538986208	00201067	PINOSYLVIN METHYL ETHER	C15H14O2	226.28	5150-38-9	stilbene		*Pinus* spp.
735	538976416	01502243	RHAPONTIN	C21H24O9	420.42	155-58-8	stilbene	antifungal, antioxidant	*Rheum* spp. *and Eucalyptus sideroxylon*
736	538976391	01502223	RESVERATROL	C14H12O3	228.25	501-36-0	stilbene	antifungal, antibacterial	*Veratrum grandiflorum, Pinus sibirica, Vitis vinifera, Arachis hypogaea*
737	3520	00201078	VIOLASTYRENE	C17H18O3	270.33	19034-96-9	styrene		*Dalbergia miscolobium*
738	1205855	01500814	CANTHARIDIN	C10H12O4	196.20	56-25-7	terpene	irritant	active principle of cantharides and other insects
739	538977923	01500156	CAMPHOR (1R)	C10H16O	152.24	464-49-3; 76-22-2	terpene	analgesic, antiinfective, antipruritic	*Cinnamomum camphora*
740	538977631	00310030	RHODINYL ACETATE	C12H22O2	198.31		terpene		common constituent of plant essential oils
741	1204261	00300106	3-AMINO-beta-PINENE	C10H18ClN	187.71		terpene		derivative
742	538977912	00300160	3-PINANONE OXIME	C10H17NO	167.25		terpene		derivative
743	538977813	01800005	MENTHYL BENZOATE	C17H24O2	260.38		terpene		derivative
744	538986487	01504800	CHRYSANTHEMIC ACID	C10H16O2	168.24	10453-89-1	terpene	esters as insecticide	esters as constituent of pyrethrum flowers
745	1069	01500294	CINEOLE	C10H18O	154.25	470-82-6	terpene	anthelminthic, antiseptic, expectorant	*eucalyptus* and lavender oils
746	538976315	01501212	LINALOOL (+)	C10H18O	154.25		terpene		*Mentha arvensis* and related essential oils
747	538978095	01503134	MENTHOL(−)	C10H20O	156.27	1490-04-6	terpene	analgesic (topical), antipruritic agent	*Mentha piperita* and other *Mentha* spp.
748	538977942	01501132	NEROL	C10H18O	154.25	106-25-2	terpene	weak estrogen receptor blocker	neroli and bergamot oils
749	538986708	01505297	PERILLYL ALCOHOL	C10H16O	152.24	536-59-4,	terpene	antineoplastic,	*Ocimum gratissimum*

						18457-55-1		apoptosis inducer; skin irritant, LD50(rat) 2100 mg/kg po	
750	1204176	00300564	MENTHONE	C10H18O	154.25	14073-97-3	terpene		pennyroyal and peppermint oils
751	538987856	01505810	PEONIFLORIN	C23H30O11	482.49	23180-57-6	terpene	antiinflammatory, antispasmodic, antihypertensive, antidiuretic	*Peonia* spp.
752	538977624	00310019	CHRYSANTHEMIC ACID, ETHYL ESTER	C12H20O2	196.29		terpene	insecticide	pyrethrum flowers
753	1204780	00300566	CHRYSANTHEMYL ALCOHOL	C10H18O	154.25	5617-92-5	terpene		reduction product of pyrrethrin constituent
754	1210903	01502101	PERILLIC ACID (−)	C10H14O2	166.22	7694-45-3	terpene	inhibits posttranslational cys isoprenylation, blocks G-protein	Salvia dorisiana
755	21279	01504167	URSINOIC ACID	C15H16O5	276.29	30265-59-9	triterpene		*Angelica ursina*
756	538978234	01504073	UVAOL	C30H50O2	442.73	545-46-0	triterpene	antineoplastic	*Arctostaphylos* spp., *Leucothoe keiskei, Crataegus cuneata, Osmanthus fragrans, Ilex latifolia*
757	1206003	01500815	BETULIN	C30H50O2	442.73	473-98-3	triterpene		*Betula* spp.
758	538978138	01504081	BETULINIC ACID	C29H46O3	442.69	472-15-1	triterpene	antineoplastic	*Betula* spp.
759	670	00102058	OLEANOLIC ACID ACETATE	C32H50O4	498.75	4339-72-4	triterpene		birch bark
760	538976379	00307045	11-OXOURSOLIC ACID ACETATE	C32H48O5	512.74		triterpene		Bursera delpechiana
761	538978140	01504082	DIHYDROCELASTROL	C29H40O4	452.64		triterpene		celastrol derivative
762	538977898	00380004	DIHYDROCELASTRYL DIACETATE	C33H44O6	536.72		triterpene	chaperone stimulant	celastrol derivative
763	538977720	01504181	PRISTIMERIN	C30H40O4	464.65	1258-84-0	triterpene	antineoplastic, antiinflammatory	*Celastrus* and *Maytenus* spp.
764	538986674	01505250	MADECASSIC ACID	C30H48O6	504.71	18449-41-7	triterpene	wound healing	*Centella asiatica*
765	427	00100551	FRIEDELIN	C30H50O	426.73	559-74-0	triterpene		*Ceratopetalum apetalum D. Don, Cunoniaceae*
766	538978105	01504016	HEDERAGENIN	C30H48O4	472.71	465-99-6	triterpene		*Clematis, Hedera* spp.
767	538985671	01500990	ENOXOLONE	C30H46O4	470.70	471-53-4	triterpene	antitussive, antiinflammatory, antibacterial	derivative

768	538986268	01504207	PRISTIMEROL	C30H42O4	466.67		triterpene		derivative of pristimerin
769	538988029	01505175	ASIATIC ACID	C30H48O5	488.71	464-92-6	triterpene	wound healing, experimental carcinogen	*Dipterocarpus pilosus,Dryobalanops aromatica*
770	538978108	01800123	ECHINOCYSTIC ACID	C30H48O4	472.71	510-30-5	triterpene		*Echinocystis* spp.
771	538978096	01500989	18alpha-GLYCYRRHETI NIC ACID	C30H46O4	470.70		triterpene	antiinflammatory	epimer of aglycone Glycyrrhiza glabra
772	538976378	00307043	DEHYDRO (11,12)URSOLIC ACID LACTONE	C30H46O3	454.70		triterpene		*Eucalyptus* spp.
773	538985957	00201697	EUPHOL	C30H50O	426.73	514-47-6	triterpene		*Euphorbia* spp.
774	43	00100552	beta-AMYRIN ACETATE	C32H52O2	468.77	1616-93-9	triterpene		latof various species of rubber tree
775	715	00100550	OLEANOIC ACID	C30H48O3	456.72	508-02-1	triterpene		leaves of *Olea europea* and *Viscum album L.*
776	538976327	00240871	OLEANANOIC ACID ACETATE	C32H52O4	500.77		triterpene		*Machaerium kuhlmannii* and *Helichrysum chrysargyrum*
777	538977791	01800031	URSOLIC ACID	C30H48O3	456.72	77-52-1	triterpene	diuretic, antineoplastic, antiulcer	*Rhododendron* spp., *Epigaea asiatica*, surface wax of fruits
778	538987988	01505133	SUMARESINOLIC ACID	C30H48O4	472.71	559-64-8	triterpene		*Styrax benzoin, Enkianthus campanulatus,Orthopter ygium huancuy*
779	2795	01800055	ZEORIN	C30H52O2	444.75	22570-53-2	triterpene		various lichens (e.g.,*Anaptychia* spp.) and *Iris missouriensis* roots
780	538977541	00100360	beta-AMYRIN	C30H50O	426.73	559-70-6	triterpene		widespread in plants
781	538976362	00240470	LANOSTEROL	C30H50O	426.73	79-63-0	triterpene		wool fat of sheep
782	538988005	01504017	SAPINDOSIDE A	C41H66O12	750.98	27013-91-8	triterpene glycoside		*Anemone coronaria, Sapindus, Hedera, Astrantia* spp.; alpha-hederin
783	1210741	01500991	GLYCYRRHIZIC ACID	C41H60O16	808.93	1405-86-3	triterpene glycoside	11beta-hydroxystero id dehydrogenase inhibitor, antiinflammatory, expectorant, antihaemorrhagic,	*Glycyrrhiza glabra*

								anti-HIV	
784	538977509	01504030	beta-ESCIN	C55H86O24	1131.28	6805-41-0	triterpene glycoside	membrane permeabilizer	principal saponin *Aesculus hippocastanum*
785	538978129	01504059	CHRYSANTHELLIN A	C58H94O25	1191.38	73039-13-1	triterpene glycoside		saponin (sugar sequences tentative)
786	1207086	00201538	DECAHYDROGAMBO GIC ACID	C38H54O8	638.85		xanthone		derivative
787	538977516	00300549	ACETYL ISOGAMBOGIC ACID	C40H46O9	670.81		xanthone		derivative
788	1207034	00201540	TETRAHYDROGAMBO GIC ACID	C38H48O8	632.80		xanthone		derivative
789	1207087	00201524	DIHYDROGAMBOGIC ACID	C38H46O8	630.79		xanthone		derivative
790	538986153	10100009	DIMETHYL GAMBOGATE	C40H48O8	656.82		xanthone		derivative of gambogic acid
791	538977713	00201522	GAMBOGIC ACID AMIDE	C38H45NO7	627.78		xanthone	caspase inhibitor	derivative of Gambogic acid
792	538978104	01504015	alpha-MANGOSTIN	C24H26O6	410.47	6147-11-1	xanthone		*Garcinia mangostana, Hydnocarpus octandra, H venenata*
793	538988148	01505481	beta-MANGOSTIN	C25H28O6	424.50	20931-37-7	xanthone		*Garcinia mangostana*
794	1201332	00200007	GAMBOGIC ACID	C38H44O8	628.77	2752-65-0	xanthone	antiinflammatory, cytotoxic, inhibits HeLa cells in vitro; LD50(rat) 88 mg/kg ip	*Garcinia morella, G hanburyii*
795	1207063	00201539	GARCINOLIC ACID	C38H46O9	646.78		xanthone		*Garcinia* spp.; also hydrolysis product of gambogic acid
796	538986693	00200523	XANTHONE	C13H8O2	196.21	90-47-1	xanthone		gentian and other flowers
797	538977576	00240736	2-METHOXYXANTHO NE	C14H10O3	226.23	1214-20-6	xanthone		*Mammea* and *Keilmeyera* spp.
798	538977574	00240651	2-HYDROXYXANTHO NE	C13H8O3	212.21		xanthone		*Mammer* and *Kielmeyera* spp.
799	538987989	01505134	MANGIFERIN	C19H18O11	422.35	4773-96-0	xanthone	MAO inhibitor, immunostimulant	*Mangifera indica*, Iris &*Salacia* spp., Aphloia, Athyrium, Anemarrhena, *Belamcanda chinensis, Hedysarum ussuriense*
800	538977572	00240642	METHYLNORLICHEX	C15H12O5	272.26		xanthone		

ANTHONE
